# A systematic investigation into the reliability of inter-temporal choice model parameters

**DOI:** 10.3758/s13423-022-02241-7

**Published:** 2023-03-06

**Authors:** Timothy Ballard, Ashley Luckman, Emmanouil Konstantinidis

**Affiliations:** 1 University of Queensland, School of Psychology, Brisbane, Australia; 2 University of Exeter Business School, Devon, UK; 3 University of Warwick, Psychology, Coventry, UK

**Keywords:** Inter-temporal choice, Delay discounting, Parameter recovery, Measurement, Computational modeling

## Abstract

Decades of work have been dedicated to developing and testing models that characterize how people make inter-temporal choices. Although parameter estimates from these models are often interpreted as indices of latent components of the choice process, little work has been done to examine their reliability. This is problematic because estimation error can bias conclusions that are drawn from these parameter estimates. We examine the reliability of parameter estimates from 11 prominent models of inter-temporal choice by (a) fitting each model to data from three previous experiments with designs representative of those typically used to study inter-temporal choice, (b) examining the consistency of parameters estimated for the same person based on different choice sets, and (c) conducting a parameter recovery analysis. We find generally low correlations between parameters estimated for the same person from the different choice sets. Moreover, parameter recovery varies considerably between models and the experimental designs upon which parameter estimates are based. We conclude that many parameter estimates reported in previous research are likely unreliable and provide recommendations on how to enhance the reliability of inter-temporal choice models for measurement purposes.

A fundamental feature of many decisions we face is the trade-off between short-term and long-term consequences. For example, the decision to attend university, eat healthy food, or to quit smoking, all offer benefits that emerge over the longer term, but also entail more immediate challenges. These types of decisions are referred to as *inter-temporal choices*, which involve deciding between options with consequences that occur at different points in time (for reviews see: Doyle, 2013; ; Green & Myerson, 2004; ; Lempert & Phelps, 2016; ; Loewenstein, Rick, & Cohen, 2008; ; Rung & Madden, 2018; ; Scholten & Read, 2010) The majority of inter-temporal choice studies have examined time preferences by asking participants to choose between monetary choice options (usually two) which vary along two dimensions: amount of money and time of receipt. A choice then involves trade-offs between options that offer relatively *smaller* rewards but are received *sooner* (*smaller-sooner* option: SS) against relatively larger rewards which are to be received later in the future (*larger-later* option: LL): for example, $100 *Today* (SS option) or $150 in *3 Months* (LL option).

Inter-temporal choice has received widespread interest across many different sub-disciplines in psychology including cognitive (Dai, Pleskac, & Pachur, 2018; Zhao, Diederich, Trueblood, & Bhatia, 2019), organizational (Ballard, Vancouver, & Neal, 2018; Brodsky & Amabile, 2018), social (Trope & Liberman, 2003; Woolley & Fishbach, 2018), developmental (Liu, Gonzalez, & Warneken, 2019; Sparrow & Spaniol, 2018), health (Berkman, 2018; Muñoz Torrecillas, Cruz Rambaud, & Takahashi, 2018), and clinical psychology (Todokoro et al., 2018; Vanyukov et al., 2016), as well as in the neuroscience (Achterberg, Peper, Duijvenvoorde, van Mandl, & Crone, 2016; Gluth, Hotaling, & Rieskamp, 2017), economics (Carvalho, Meier, & Wang, 2016; Castillo, Jordan, & Petrie, 2019), and management (Crilly, 2017; Cubitt et al., 2018)literatures. The core assumption (and fundamental empirical result) underlying theories of inter-temporal choice is that people *discount* the value of future outcomes. As a result, the subjective value of an outcome is a decreasing function of the amount of time before which the outcome is experienced. This phenomenon has been referred to as temporal, time, or delay discounting.

The canonical and rational framework (or model) of temporal discounting is exponential discounting whereby the value of a future reward decreases with a constant proportion (for more details see Section on Exponential Discounting). However, human behaviour has been found to be largely inconsistent with exponential discounting. A common finding is that of hyperbolic discounting (or decreasing impatience), whereby people discount future rewards less strongly as time passes. Decades of psychological research in inter-temporal choice has identified a) that people’s observed behavior deviates from rational theories (e.g., exponential discounting) in numerous ways and b) the psychological and cognitive determinants of how people represent time and make choices that involve delayed outcomes. In particular, the development and testing of computational models has accelerated our understanding of how factors such as pleasure (gains), pain (losses), time and uncertainty, influence people’s inter-temporal choices (e.g., Ainslie, 1975; Ebert & Prelec, 2007; Gonzalez-Vallejo, 2002; Killeen, 2009; Loewenstein & Prelec, 1992; Marzilli Ericson, White, Laibson, & Cohen, 2015; Samuelson, 1937).

More recently, the parameters of computational models of inter-temporal choice have become the focus of investigation. These models have been used as measurement tools, whereby parameter estimates are interpreted as indices of latent components of the choice process. For example, estimates of the discount rate parameter of the exponential and hyperbolic models, which reflects the rate at which future outcomes are discounted, is often used to measure individual differences in time preferences^1^ (e.g., impulsively or impatience (Kirby, Petry, & Bickel, 1999); (Muñoz Torrecillas, Cruz Rambaud, & Takahashi, 2018)). Differences in discount rate parameter estimates have been explored between people of different age groups (Samanez-Larkin et al., 2011), clinical populations (Cheng & González-Vallejo, 2014), and across a variety of experimental manipulations (Haushofer et al., 2013; Li et al., 2018; Liu, Gonzalez, & Warneken, 2019; Marzilli Ericson, White, Laibson, & Cohen, 2015). For instance, the discount rate parameter from the hyperbolic discounting model is frequently used to compare time preferences in substance abusers and non-clinical populations (for a review see MacKillop et al., 2011, such as in Cheng and González-Vallejo (2014) where discount rates were found to be higher for heroin users than a matched control group, indicating more impatience for the users group. Parameter values from similar models, such as the hyperboloid model, have also been used to measure changes in behaviour within an individual, such as changes in time preferences due to the magnitude of the outcome (Myerson & Green, 1995).

Parameter estimates from inter-temporal choice models are also often compared with process measures, such as measures of attention (Amasino, Sullivan, Kranton, & Huettel, 2019; Fisher & Rangel, 2014). For instance, using eye-tracking to measure visual attention, Fisher & Rangel found that the hyperbolic discount rate parameter correlates with the amount of time people spend looking at information about delays, suggesting that impatience, as measured by discounting, may be caused by attentional biases. Similarly correlations between parameter estimates and measures of neural activity are commonly used to make inferences about how these processes play out in the brain (e.g., Bos, van den Rodriguez, Schweitzer, & Mcclure, 2014; Kable & Glimcher, 2007; Liu, Feng, Wang, & Li, 2012; Marco-Pallarés, Mohammadi, Samii, & Münte, 2010). Even when the parameter values are not the primary focus of the research, it has become common practice to report their estimates and interpret them as meaningful. For instance, Marzilli Ericson et al., (2015) report how the weighting parameters (i.e., representing the degree of importance placed on numerical values for outcomes and delays) of their ITCH model vary across different framing conditions and link this to the salience of different attributes or types of comparisons within those conditions.

The tacit assumption implied from the research described above is that estimates of model parameters reliably capture meaningful psychological content. Despite the increasing emphasis on interpreting parameter estimates, however, we know of very little research that has examined the reliability associated with inter-temporal choice models’ parameter estimates. This is important, because estimation error can systematically bias conclusions that are drawn from model parameters. Without first establishing that a model’s parameters can be reliably estimated, it is difficult to know whether meaningful conclusions can be made based on parameter estimates (Heathcote, Brown & Wagenmakers 2015 Wilson and Collins, 2019).

In this paper, we examine the estimation properties of 11 prominent models of inter-temporal choice (see Table 1). We do so by conducting parameter recovery simulation analyses, separately for experimental data from three different studies. In each study, we simulate data from a prototypical inter-temporal choice experiment using a range of parameter values for each model. We then fit each model to the simulated datasets and examine the extent to which the data-generating parameter values are recovered. To anticipate our main results, we find that the models vary widely in their parameter recovery, with some models demonstrating excellent recovery and others demonstrating very poor recovery. Recovery also depends on the design of the study that is used to estimate the parameters. In study 3, we also show that parameter values for the same person differ depending on the choice sets used, suggesting a lack of consistency in their estimates. We conclude that researchers should use extreme caution when interpreting the parameter estimates from certain models of inter-temporal choice, especially when parameter estimates are based on data from certain sets of items. We also make recommendations regarding which approach might offer more recoverable estimates for researchers wishing to use these models for measurement purposes. The results and recommendations of the current research not only will inform the use of such models in future studies of inter-temporal choice, but have the potential to (re)shape the methods and experimental designs that are used to assess people’s preferences with delayed rewards.

**Table 1:** Summary of models examined

Model	Function(s)	
Exponential	$V_{i} = x_{i} e^{-kt_{i}}$	(1)
	*p*_*LL*_ = *L*[*σ*(*V*_*LL*_ − *V*_*SS*_)]	(2)
Hyperbolic	$V = \frac {x_{i}}{1+k t_{i}}$	(3)
Double exponential	$V_{i} = x_{i}[\omega e^{-\beta t_{i}} + (1-\omega ) e^{-\delta t_{i}}]$	(4)
Generalized hyperbolic	$V_{i} = \frac {x_{i}}{(1+k {t_{i}^{s}})}$	(5)
Hyperboloid	$V_{i} = \frac {x_{i}}{(1+k t_{i})^{s}}$	(6)
Generalized hyperbola	$V_{i} = \frac {x_{i}} {(1 + \alpha t_{i})^{\beta / \alpha }}$	(7)
Constant sensitivity	$V_{i} = x_{i} e^{-(\alpha t_{i})^{\beta }}$	(8)
Additive utility	$V_{i} = x_{i}^{\alpha } - \lambda t_{i}^{\beta }$	(9)
Proportional difference	$d = \frac { \max \limits {(|x_{LL}|,|x_{SS}|)} - \min \limits {(|x_{LL}|,|x_{SS}|)}}{\max \limits {(|x_{LL}|,|x_{SS}|)} } - \frac { \max \limits {(|t_{LL}|,|t_{SS}|)} - \min \limits {(|t_{LL}|,|t_{SS}|)}}{\max \limits {(|t_{LL}|,|t_{SS}|)} } $	(10)
	*p*_*LL*_ = Φ[*σ*(*d* − *δ*)]	(11)
ITCH	$p_{LL} = L[\beta _{1} + \beta _{xA} (x_{LL} - x_{SS}) + \beta _{xR}(\frac {x_{LL} - x_{SS}}{x^{*}}) + \beta _{tA}(t_{LL} - t_{SS})$	
	+ $\beta _{tR}(\frac {t_{LL} - t_{SS}}{t^{*}})] $	(12)
Trade-off	$ v_{i} = \frac {1}{\gamma }\log (1+\gamma x_{i}) $	(13)
	*Q*_*v*_ = *v*_*LL*_ − *v*_*SS*_	(14)
	$ w_{i} = \frac {1}{\tau }\log (1+\tau t_{i}) $	(15)
	$ Q_{w} = \frac {\kappa }{\alpha }\log [1+ \alpha (\frac {w_{LL} - w_{SS}}{\theta })^{\theta }] $	(16)
	$ p_{LL} = \frac {Q_{v}^{1/\epsilon }}{Q_{v}^{1/\epsilon } + Q_{w}^{1/\epsilon }} $	(17)

Note: In the above equations, *x*_*i*_ and *t*_*i*_ represent the monetary outcome and temporal delay associated with option *i* respectively. *L*(*x*) represents the CDF of the standard logistic distribution evaluated at *x*, Φ(*x*) represents the CDF of the standard normal distribution evaluated at *x*, $\log (x)$ represents the natural logarithm of *x*. For models with no likelihood function explicitly specified (all except the ITCH, proportional difference, and tradeoff models), we assumed the likelihood function given in Eq. 2. To facilitate estimation of the Proportional Difference model, we reparameterized the likelihood function for this model so that higher values of *σ* produced more deterministic decisions. The value function for the Trade-off model differs for negative payoffs (see Scholten et al., 2014). However, since there were no negative payoffs in any of the three experiments presented here, only the value function for positive payoffs is shown in the table

## Effects and models of inter-temporal choice

In this section, we provide an overview of the inter-temporal choice models that we examine in this research. We consider 11 prominent models from the literature, which differ in two key ways: Firstly, they differ in which behavioral effects/regularities they predict, and secondly in the theoretical assumptions they make about the processes which generate these effects. Therefore, depending on the effects they consider to be reliable and the theoretical accounts they consider plausible, different researchers may use different models to measure individual time preferences. In this section we highlight the differences between the 11 models with respect to how they account for (or not) four behavioral regularities; the *common difference effect*, the *magnitude effect*, the *delay duration effect*, and *interval effects*. We address these effects because they are important for describing the motivation behind certain models. However, this is not intended to be an exhaustive list of inter-temporal choice phenomena.

### Inter-temporal choice effects

The *common difference effect* refers to the tendency for people’s preferences to change with time (Dai & Busemeyer, 2014; Scholten, Read, & Sanborn, 2014). Normatively, behavior should be consistent over time. This means, for instance, if one prefers to receive $120 in two years compared to receiving $100 in one year, then they should also prefer $120 in one year to $100 today. The previous example suggests that as long as the temporal distance of 1 year is preserved across different choice options (with the same monetary amounts), then the option that is further delayed should always be selected. However, people often switch preferences between these two choices, preferring the larger-later option in the first choice (i.e., $120 in 2 years; a common delay of 1 year in both options), whereas they choose the smaller-sooner option in the second choice (i.e. $100 today; Weber & Chapman, 2005). More generally, adding a common delay to both options tends to make people more patient. This effect has also been referred to as the immediacy effect (Keren & Roelofsma, 1995), delay effect (Scholten & Read, 2006), dynamic inconsistency (Thaler, 1981), or hyperbolic discounting (Kirby & Herrnstein, 1995).

The *magnitude effect* refers to the tendency for preferences to change with the absolute value of the outcomes. Normatively, increasing the outcome magnitude of both outcomes by a common multiplicative factor should not influence preferences. According to discounted utility models (explained in detail below) for example, if both outcomes are multiplied by a common factor (e.g., 10) then the discounted utility of both options will also be multiplied by that factor, and therefore the preferred option should not change. Experimental evidence, however, suggests that people tend to be more patient (i.e., more likely to pick the larger-later option) when the magnitude of the outcomes is increased (Thaler, 1981; Vincent, 2016).

The *delay duration effect* refers to the observation that people tend to prefer the larger-later option less when the time delays of both options are multiplied by a constant greater than one (Dai & Busemeyer, 2014). Unlike the previous two effects, this behaviour is consistent with normative accounts. Multiplying the delays in this fashion increases the delay of the larger-later option more than that of the smaller-sooner, leading to a relatively greater increase in the amount the larger-later option is discounted.

Finally, we consider two *interval effects*. The first of these interval effects is called *sub-additivity* and refers to the finding that when discounting is assessed over a whole interval (e.g., 1 year) the observed rate of discounting is lower than when it is assessed over sub-intervals that make up that year (e.g., over each of the 12 months in the year). The second interval effect, *super-additivity* is the reverse, with more discounting over the whole interval than would be expected based on discounting of the subintervals (Scholten & Read, 2010). Preferences appear to shift from showing sub-additivity to super-additivity as the interval between outcomes decreases and/or as the magnitude of the outcome increases (Scholten & Read, 2010; Scholten, Read, & Sanborn, 2014).

### Discounted utility models

One of the earliest models proposed to account for inter-temporal choice behavior is the exponential discounting model (Samuelson, 1937). This model, and the majority of those we examine, are discounted utility models, which assume that people decide between inter-temporal prospects as if they are discounting the value (or utility) of each option based on how delayed it is, then choosing the option with the highest discounted utility. In the exponential discounting model the rate at which outcomes are discounted is stable, that is, for each unit of time an outcome is delayed it loses some stable proportion of its (remaining) value. Formally, this is captured by the exponential discounting function (Table 1, Eq. 1), where the outcome of an option is multiplied by a discount term, which is an exponential function of the time at which it will be received. In the exponential discounting model, *k* is a discount rate parameter, which measures differences in the rate at which value is lost (i.e., discounting occurs), with larger values of the parameter indicating greater discounting.

Equation 1 specifies how the discounted utility of each option is calculated. The model is then applied to choice data by mapping utilities to choice actions. The standard economic approach is a *deterministic* mapping, which assumes that people always choose the option with the highest discounted utility (regardless of the size of the utility difference). However, decades of research on the nature of preferential choice (e.g., inter-temporal choice and risky choice) has shown that people’s choices are better aligned with “noisy” and *probabilistic* preferences (Rieskamp, 2008); that is, people do not always make the same choices (e.g., maximizing some measure of subjective utility) when presented with similar choice sets, and sometimes opt for options with lower discounted utilities. Therefore, in line with much of the literature (e.g., Dai & Busemeyer, 2014), we assume in the current work that the probability of choosing the larger-later option is a logistic function of the difference in the discounted utilities of the two options (Table 1, Eq. 2). In the logistic function, *σ* is a free parameter governing how deterministic a person is, with larger values indicating a steeper logistic function and therefore more deterministic decisions. This choice model can also be conceptualised as people choosing in a deterministic manner, but having logistically distributed noise (i.e., mistakes), with mean 0 and scale $\frac {1}{\sigma }$ in the calculation of the utility difference. For consistency, we will use this choice function for all 7 of the discounted utility models we consider.

Although influential, the exponential model, due to its stable discount rate, does not account for the common difference effect, which implies lower discount rates for the more distant time period (1 to 2 years) than the more proximal period (0 to 1 years). The other 7 discounted utility models we consider were all proposed, in part, to capture the common difference effect, albeit in different ways. Below we categorise these models based on how they produce this effect. It should be noted that all of these discounted utility models, including the exponential, are able to account for the delay duration effect, and with one exception, unable to account for the magnitude effect in their most common forms.^2^

#### Decreasing discount rates with time

A simple way to model the common difference effect is to build a model with discount rates that decrease for more temporally distant time points, rather than remaining stable as in the exponential discounting model. This is how the *Hyperbolic discounting* model produces the common difference effect (Mazur, 1987). As the name suggests, hyperbolic discounting assumes that the discount function is a hyperbolic function of time, rather than an exponential function (see Table 1, Eq. 3). In a hyperbolic model, rather than outcomes losing a stable proportion of their remaining value for each unit of time they are delayed, instead they lose a decreasing proportion of their remaining value. In Eq. 3, the *k* parameter is similar to the *k* parameter in the exponential discounting function (Eq. 1). This parameter determines the rate at which discounting occurs, with higher values of the *k* parameter indicating higher discounting.

The *Double-Exponential* model produces a similar pattern of decreasing discount rates by assuming that people apply two separate discount rates to each choice option, given by the *β* and *δ* parameters in Eq. 4. The discounted value of an option is the weighted sum of the discounted values produced by these two rates (Mcclure, Ericson, Laibson, Loewenstein, & Cohen, 2007), with the parameter *ω* (0 < *ω* < 1) determining the relative weight given to each value. Mcclure et al., argue that these two separate discount rates may be the result of different brain systems being involved in the decision-making process, with more future oriented/patient systems producing lower discount rates, *δ*, than more present focused impatient systems, *β*. Due to the shape of the exponential function, averaging across two or more different exponential functions produces a pattern of decreasing discount rates, similar to the hyperbolic function.

#### Diminishing sensitivity to time

The *Hyperboloid* (Myerson & Green, 1995), *Generalized Hyperbolic* (Rachlin, 2006) and *Generalized Hyperbola* (Loewenstein & Prelec, 1992) models extend the baseline Hyperbolic model by including a second mechanism (on top of hyperbolic discounting) which can independently produce the common difference effect. All three models assume that discounting does not occur over objective time, but instead over some form of subjective or psychological time. If psychological time is assumed to be a concave function of objective time, then people will exhibit diminishing sensitivity to time. That is, the difference between 1 year and 2 years from now will be treated as shorter than the difference between now and 1 year from now, despite both objectively being one year apart. If discounting then occurs over this perceived time, the common difference effect will occur, as adding a common delay will shorten the perceived distance between the two outcomes, and will therefore reduce the amount of extra discounting the larger-later option undergoes relative to the smaller-sooner.

The Generalized Hyperbolic model (Table 1, Eq. 5) incorporates this notion by assuming that psychological time is a power function of objective time, with *s* determining the curvature of the relationship, where 0 < *s* < 1 produces a concave relationship and *s* > 1 a convex relationship (i.e., increasing sensitivity to time). The Hyperboloid and Generalized Hyperbola models (Eqs. 6 and 7, respectively) use a slightly different form to produce diminishing sensitivity to time, with the entire denominator taken to the power, rather than just time. However, this will similarly produce a concave relationship between objective and subjective time when 0 < *s* < 1 (Eq. 6) or *β* < *α* (Eq. 7), and therefore similar effects. The Hyperboloid and Generalized Hyperbola can be considered different parameterizations of the same underlying model, with *k* = *α* and $s = \frac {\beta }{\alpha }$.

Diminishing sensitivity to time can also produce the common difference effect without the need for hyperbolic discounting. The *Constant Sensitivity* model (Table 1, Eq. 8) (Ebert & Prelec, 2007) produces the effect by assuming that an exponential discount rate is applied over psychological time, rather than objective time. As with the Generalized Hyperbolic model, the Constant Sensitivity model assumes that psychological time is a power function of objective time, with *β* in Eq. 8 having a similar interpretation to *s* in Eq. 5, and *α* a similar role to *k* in Eq. 1.

The final discounted utility model we consider (*Additive Utility* model; Table 1, Eq. 9) also explains the common difference effect through diminishing sensitivity to time, by assuming that the discount rate is based on power-transformed time rather than objective time. However, its conceptualisation of how discounting occurs is very different, and this allows it to produce effects that the other models do not. In the previous seven discounted utility models, the absolute amount of value that an outcome loses per unit of time delay is a function of the outcome value, as shown by the discount rate being multiplied by the value (see Eqs. 1 & 3–8). In the Additive Utility model (Killeen, 2009) the amount of discounting is independent of the value of the outcome; Instead the amount of utility (or value) an outcome loses due to being delayed depends only on the psychological/subjective length of the delay and the discount rate parameter, *λ* (Eq. 9). This allows the Additive Utility Model to capture the magnitude effect. Multiplying the magnitude of the outcomes by a constant increases the relative attractiveness of the larger-later option, and this increase in attractiveness is not offset by any increase in the effect of delay discounting. As a result, increases in magnitude shift preferences toward the larger-later option.

Although unrelated to the common difference and magnitude effects, the Additive Utility model also differs from the other discounted utility models as it assumes that the utility of an outcome is not its objective value, but rather a power transformation thereof, with *α* determining the curvature of the function. A similar utility function could be added to the other discounted utility models (see (Dai & Busemeyer, 2014). However, the canonical versions of these models do not include such a transformation and typical applications of these models assume a linear mapping between objective value and utility. The empirical evidence also suggests that this mapping is generally closer to linear for inter-temporal choices than it is for other domains where non-linear transformations are standard, such as risky decision making (Abdellaoui, Bleichrodt, l’Haridon, & Paraschiv, 2013; Cheung, 2020). As the goal of our analysis is to examine the parameter estimates in contexts in which the models are typically applied, we only apply transformations of the objective value where specified in Table 1.^3^

### Attribute-wise models

The final three models we consider do not belong in the discounted utility category. Rather than assuming that people decide as if they calculate the discounted utility of each option separately, these models assume that people compare *attribute* values across options and decide by weighting the attributes against each other. In all three models presented here, it is assumed that people consider the differences in the outcomes (e.g., $200 vs. $100) and delays (e.g., 2 years vs. 1 years) between choice options, and then reach a decision by weighting these differences against each other. The exact way in which the differences in attributes are computed and weighted against each other differs between attribute-wise models.

These so-called attribute-wise models were developed in response to evidence inconsistent with the discounted utility framework and models, such as the interval effects (Scholten & Read, 2010). Process-tracing methods and measurements (e.g., eye-tracking data) provide additional support for attribute-wise models, showing that comparing choices along individual attributes (e.g., amount or delay) is a frequent strategy among participants (Amasino, Sullivan, Kranton, & Huettel, 2019; Reeck et al., 2017). The distinction between discounted utility (or alternative-wise) and attribute-wise models also parallels ongoing debates in other areas of decision making (e.g., risky choice) as to whether people use rules and heuristics based on simple attribute comparisons to make decisions, or engage in the arguably more cognitively demanding integration of information across attributes required by models such as discounted utility models (Brandstatter, Gigerenzer, & Hertwig, 2006; Gonzalez-Vallejo, 2002).

The simplest of the three attribute-wise models, the *Proportional Difference* model, assumes that people consider the proportional differences in each of the two attributes (Gonzalez-Vallejo, 2002). These proportional differences are calculated by dividing the difference in each attribute by the maximum value offered for that attribute (Table 1, Eq. 10). A decision is then made by calculating the difference between these two differences, and comparing it to a threshold value *δ*. If the difference is greater than the threshold then the larger-later option is chosen, if it is smaller the smaller-sooner is chosen. As with the discounted utility models, this decision process is assumed to be noisy (Eq. 11), although in this case with normally distributed noise. The threshold parameter, *δ*, can be considered a bias parameter, with positive values indicating a bias for choosing the smaller-sooner option and negative values the larger-later option.

By assuming that people consider the proportional difference in delay, the Proportional Difference model naturally captures the common difference effect, as adding a common delay to both options increases the maximum delay, while leaving the absolute difference in delay unchanged. Conversely, using proportional differences in amount means that the Proportional Difference model does not capture magnitude effects. The Proportional Difference model is also the only model considered here that does not capture the delay duration effect. This is because multiplying the delays by a constant has no impact on the proportional difference in delay, and therefore the Proportional Difference model predicts that doing so will have no impact on preferences.

The *Inter-Temporal Choice Heuristic* (ITCH; Marzilli Ericson et al., 2015) builds on the Proportional Difference model and assumes that people consider both proportional and absolute differences in each attribute dimension (Table 1, Eq. 12). This assumption allows the ITCH model to capture the magnitude effect, as multiplying both outcomes by a constant increases the absolute difference in outcomes by that same constant, making the larger-later option relatively more attractive. It similarly allows it to capture delay duration effects. The ITCH model differs from the Proportional Difference model by assuming that, in addition to having a bias towards choosing sooner or larger options, given by *β*_1_, participants can also vary in the amount of weight they give each of the four types of differences: absolute delays (*β*_*tA*_), relative delays (*β*_*tR*_), absolute amounts (*β*_*xA*_), relative amounts (*β*_*xR*_). It also uses the mean attribute values (rather than the maximum) as the reference point when calculating the proportional difference.

The final model we consider, the *Trade-off model* (Scholten & Read, 2010), was developed to capture the two interval effects (which the other 10 models we address here do not account for), in addition to the other three effects. The Trade-off model assumes that people make decisions by comparing the difference in the utility of the two options to the difference in the delay of the two options. As with the Additive Utility model, the Trade-off model assumes that the utility of an outcome is a concave function of the objective outcome (in this case in the log scale; Table 1, Eq. 13). Similarly, people do not consider the objective delays to the two outcomes, but rather psychologically weighted delay, which is also a log function of the objective delays (Table 1, Eq. 15). By assuming that people calculate the absolute difference in the utility of the two outcomes (Table 1, Eq. 14), the Trade-off model is able to produce the magnitude effect. By assuming that psychologically weighted delays are a concave function of objective delays, it can produce the common difference effect through diminishing sensitivity to delay. The Trade-off model produces sub-additivity and super-additivity through the weighting function applied to the difference in psychological delays (Table 1, Eq. 16). This function weights the differences in delays against the difference in amounts, with the *κ* parameter in Eq. 16 determining the relative weighting of the two, the *α* parameter determining the extent to which sub-additivity is shown, and the *𝜃* parameter the extent to which super-additivity is shown.^4^

## What makes a parameter estimate meaningful?

As seen in the previous section, each model contains parameters that are intended to map onto psychologically meaningful constructs (e.g., discount rates, perceptions of time). Estimates of these parameters can, in theory, provide insight into the underlying construct they are meant to reflect. For example, a researcher wishing to examine the weight people place on the difference in amounts might interpret the *β*_*xR*_ parameter from the ITCH model. Another researcher wishing to quantify the extent to which people demonstrate sub-additivity in a certain context might interpret the *α* parameter of the Trade-off model. But how do we ensure that model parameters are reliable representations of the latent psychological and behavioral constructs? The first step is fitting each candidate model to the data and comparing their ability to parsimoniously account for the observed patterns of behavior. The rationale is that the model that provides the best account for the data should also provide the best account for the behavior under investigation. Consequently, the calibrated/estimated model parameters should also have been “tuned” in a way that reveal and explain numerical properties of the latent construct that has been instantiated by the model and its interactions and relationships with other assumed psychological constructs (inside or outside the model scope). For example, (Green & Myerson, 2004) showed that a 2-parameter hyperbolic model provides the best account for the delayed choices of three different age groups, suggesting that discounting of delayed rewards (in this particular dataset) is best described by this functional form (as opposed to other functional forms such as exponential discounting). Green et al. compared the parameters from this model across different age groups and used these differences to draw conclusions about how discounting behavior changes with age (see also; Green, Fry, & Myerson, 1994).

Such conclusions about latent psychological processes rest on the assumption that parameter values reliably and accurately measure the construct that they are intended to measure. However, human behaviour is noisy, idiosyncratic, and adaptive. Noise may refer to anything that obstructs measurement of the intended construct. For example, in the context of inter-temporal choice, noise can be introduced: a) due to participants having weak or inconsistent preferences between choice options or b) due to poorly designed experiments (i.e., choice sets) that do not allow for participants’ true preferences to be inferred. When fitting models to behavioral data, we necessarily assume that some level of noise is reflected in our model’s parameter estimates, though what we assume to be noise will depend on the structure of the model and the processes it represents. Indeed, noise itself can be a construct of interest (Gershman & Bhui, 2020). Ideally, we want to minimize the degree of noise that intrudes into the parameter estimates, or similarly, to ensure that the model does not *over-fit* the data (see e.g., (Pitt & Myung, 2002); (Roberts & Pashler, 2000)). In other words, a near-perfect model fit of the data may not be desirable, as it may not allow for the separation of the true generating process responsible for observed behavior and noise.

Parameter non-identifiability is another problem that can obscure conclusions drawn from estimated parameter values (e.g., Bamber & van Santen, 1985; Krefeld-Schwalb, Pachur, & Scheibehenne, 2022; Moran, 2016; Spektor & Kellen, 2018). The issue of non-identifiability occurs when more than one set of parameter combinations produce identical predictions about observed behavior. In other words, there is not a unique mapping between model parameters and data, and thus observed behavior can be explained by more than one set of numerical values for the model parameters.

Parameter recovery can help identify issues with over-fitting and non-identifiability. The main objective of recovery is to establish the accuracy and consistency of model parameters estimated from (synthetic) datasets that have been produced using known parameter values (Heathcote, Loft, & Remington, 2015). The advantage of this exercise is that we already know two important characteristics about the synthetic data: the generating model and the model parameter values. With behavioral data we are unsure about both. Knowing the true model of the data and providing the model with the desired parameter values, we can then examine whether the parameters that generated the synthetic data can be recovered when the same model is fit to these synthetic data. This helps researchers to ascertain the extent to which a model’s parameters can be reliably estimated and, if so, identify designs that are appropriate for estimating them.

Parameter recovery exercises have been conducted with models from different areas in preferential choice. Such exercises are important especially when the models are used as measurement models with the parameters differentiating between groups (e.g., different age groups as in the example above) or characterizing observed behavior (e.g., level of impatience based on estimated discount rate). For example, (Nilsson, Rieskamp, & Wagenmakers, 2011) performed a parameter recovery on Cumulative Prospect Theory (CPT (Tversky & Kahneman, 1992)). The exercise showed that the loss aversion parameter which constitutes a key aspect of the model, was hard to reliably estimate and recover. Loss aversion correlated with the diminishing sensitivity parameter for losses, as both parameters capture how losses are being treated. Problems with loss aversion and other CPT parameters have also been identified elsewhere (e.g., (Broomell & Bhatia, 2014); (Krefeld-Schwalb, Pachur, & Scheibehenne, 2022)). Within the inter-temporal choice literature, previous work has highlighted problems with estimating and interpreting parameters from the hyperbolic discounting model (Vincent & Stewart, 2020; Molloy et al., 2020; Apesteguia & Ballester, 2018). However, no large scale investigation has been conducted that systematically examines parameter recovery using a broad set of models and different datasets.

Identification of potential problems with certain model parameters can also help provide solutions on how to best assess the latent construct that a certain parameter is assumed to measure. For example, loss aversion may be indeed an important element of risky choice, but the way it is implemented via the functional form of CPT makes it almost indistinguishable from other parameters and elements of the model (namely, diminishing sensitivity for losses). Certain remedies (e.g., different functional forms and constraints on the parameter values) can help solve problems with recovery performance and reduce redundancy between model parameters.

A similar issue relating to the reliability of model parameters is that of parameter consistency. The rationale is that if a parameter reflects and measures a stable individual trait, its value should correlate across different tasks exploring the same psychological construct (Konstantinidis, Speekenbrink, Stout, Ahn, & Shanks, 2014; Yechiam & Busemeyer, 2008). Theoretically, loss aversion should be a stable individual characteristic and describe behavior in similar environments or experimental tasks. For example, high correlations should be observed in the loss aversion parameter when participants making choices between risky prospects or when they evaluate individual lotteries (i.e., providing the certainty equivalent of a risky prospect).

## Overview of current research

In this research, we examine the ability of each model to account for widely observed behavioral regularities and the reliability of parameter estimates based on experimental protocols that have been used to examine these regularities. Our analysis consists of three phases. In the first phase, we perform a novel assessment of each model’s descriptive adequacy, comparing the ability of all 11 models to account for the pattern of empirical results in three prior studies that use different experimental designs (see Table 2). We then examine the consistency in each model’s parameter estimates by assessing the correlation between parameters estimated from the same individual across different experimental conditions. In the third phase, we conduct a parameter recovery analysis to examine the extent to which each model’s parameters can be identified in the designs used in each study.

**Table 2:** Summary of datasets used to assess models

Study	Original Experiment	Description	Participants	Items	Delays (SS/LL)	Magnitudes (SS/LL)
1	Read et al. (2017; Study 2)	Used Kirby monetary choice questionnaire (Kirby et al., 1999)	262	27	immediate / 7-186 days	11-80 / 25-85 (GBP)
2	Konstantinidis et al. (2020; inter-temporal choice portion of Experiment 1)	Factorially manipulated delay and magnitude	38	380	immediate / 2-38 months	100 / 120-500 (AUD)
3	Dai and Busemeyer (2014; Experiment 3)	Used adjustment procedure which tailored items to participants to induce specific effects	37	300	2-40 / 4-200 days	2-40 / 4-80 (USD)

In order to conduct a rigorous assessment of the models, we utilize items and data from three prior studies that are representative of the types of experiments that are commonly used to examine models of inter-temporal choice (see Table 2). In Study 1, we use data from an experiment by Read et al., (2017; Study 2) which employed the Kirby monetary choice questionnaire (Kirby, Petry, & Bickel, 1999), one of the most commonly used sets of choice items for studying inter-temporal choice (Duckworth & Kern, 2011; MacKillop et al., 2011; Rung & Madden, 2018). This choice set is designed to measure hyperbolic discount rates (*k*, Eq. 3) and has been used to study time preferences in a variety of domains, such as studies of addiction (Kirby & Petry, 2004; Kirby et al., 1999; MacKillop et al., 2011), education achievement (Duckworth & Seligman, 2005; 2006), neuroimaging (Contreras-Rodríguez et al., 2015; MacKillop et al., 2012; Monterosso et al., 2007), self-control (Duckworth & Kern, 2011), field studies (Chabris, Laibson, Morris, Schuldt, & Taubinsky, 2008), and experimental manipulations of discounting (Bulley et al., 2019; Read, Olivola, & Hardisty, 2017; Rung & Madden, 2018), amongst others (Foxall, Doyle, Yani-de, & Wells, 2011; Housden, O’sullivan, Joyce, Lees, & Roiser, 2010; Tate, Tsai, Landes, Rettiganti, & Lefler, 2015). This choice set contains 27 items which pit a larger-later option against an option that offers an immediate reward of between $11 and $80. The delay for the larger-later option ranges from 7 to 186 days and the reward associated with this option ranges from $25 to $85. These items are often analysed as 3 sets of 9 items, with the magnitude of the outcomes involved increasing across the sets. Within each set the items were designed so that the delay at which a participant switches from preferring the larger-later to smaller-sooner option can be used to estimate the participant’s hyperbolic discount rate.

Although the Kirby et al. items are among the most commonly used, it may be that a larger set of items is needed to obtain reliable parameter estimates from certain models. In Studies 2 and 3, we therefore used data from studies which employed much larger choice sets. In Study 2, we use data from an experiment by Konstantinidis et al., (2020; the inter-temporal choice component of Experiment 1), which provided a more fine-grained manipulation of delay duration and reward magnitude. This experiment consisted of 380 inter-temporal choices, in which participants had to choose between $100 now and $X in *D* months, where $X varied between $120 and $500 in $20 increments and *D* varied between 2 months and 38 months in 2-month increments. Participants were presented with every combination of amount and delay once. In Study 3, we use data from an experiment that was designed to elicit three specific effects: the common difference, magnitude, and delay duration effects. In this experiment, originally reported by Dai and Busemeyer (2014); Experiment 3,^5^ participants chose between two delayed rewards and different inter-temporal choice options were generated for each participant. They used an adjustment procedure to generate choice sets that were tailored to each participant. The goal was to produce items that would be most suitable for eliciting each of the three effects for that participant. Each participant made 300 inter-temporal choices (100 for each effect). For the items relating to the delay duration effect, the larger-later options always had a delay that was twice as long as the delay for the smaller-sooner options. For the items relating to the common difference effect, the larger-later delay always exceeded the smaller-sooner delay by a constant amount (30 days). For both the delay duration and the common difference effect, the shorter delays ranged between 2 and 40 days. For the items relating to the magnitude effect, the larger-later reward amount was always twice as much as the smaller-sooner reward, with the smaller-sooner rewards ranging from $2 to $40. All the data and code necessary to conduct the analyses can be found at https://osf.io/5phxn/. These analyses were not pre-registered.

## Descriptive adequacy

We began by fitting each model to the datasets described above and examining the extent to which each model accounts for the patterns of inter-temporal choices in each experiment. This also allowed us to ascertain the most plausible parameter values for each model, so that the parameter recovery simulations would be based on parameter values that are within the range of those observed in these experimental settings. We fit each model using a hierarchical Bayesian framework, which assumes that parameters varied across participants but were drawn from common population distributions. The priors for each model are shown in Appendix A.

The posterior distributions were estimated using the No-U-Turn Sampler (Hoffman & Gelman, 2014) as implemented by the Stan platform (Carpenter et al., 2017). For each model, we ran four chains. Each chain had a burn-in period of 2000 samples. After burning-in, each chain produced a further 2000 samples. The analysis was therefore based on 8000 samples (e.g., 4 chains × 2000 post burn-in samples per chain). These settings are typical for model fits using Stan as its sampler produces a relatively high number of effective samples per iteration and therefore typically requires far fewer samples than other algorithms to reach convergence.

**Fig. 1:**
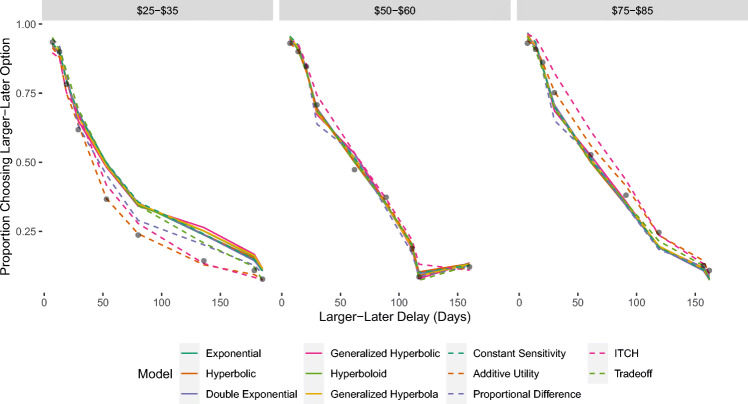
Proportion of choices in which the larger-later option was chosen in the experiment used for Study 1. Observed proportions are represented by black dots. Predictions were generated based on the mean of the posterior predictive distribution. The three panels represent differences in the magnitude of the reward for the larger-later option

Figure 1 shows the fit of the models to the data that was used for Study 1. As can be seen, participants in this experiment became less likely to select the larger-later option as the delay (represented on the x-axis) increased. All 11 models accurately capture this qualitative trend, though many models underestimate the strength of the effect when the magnitude of the larger-later option (represented as the different panels) is smaller. We used the WAIC (Watanabe, 2013) to measure the predictive accuracy of the models (see Table 3). The model that performed best according to this measure was the Additive Utility model.

**Table 3:** Results of model comparisons for Studies 1, 2, and 3

	Study 1			Study 2			Study 3		
	WAIC	SE	Rank	WAIC	SE	Rank	WAIC	SE	Rank
Exponential	3140.03	88.49	8	7678.12	134.06	8	10082.57	121.12	9
Hyperbolic	3138.66	88.45	7	7677.44	131.59	7	9605.51	119.56	7
Double exponential	3102.99	88.58	3	7257.16	131.35	4	8397.71	125.71	3
Generalized hyperbolic	3106.46	87.51	6	7256.28	131.9	3	8397.21	125.25	2
Hyperboloid	3104.92	87.94	4	7329.27	131.78	6	8675.39	123.48	5
Generalized hyperbola	3106.11	87.95	5	7328.88	131.8	5	8675.61	123.48	6
Constant sensitivity	3158.26	88.46	9	7243.11	131.67	1	8455.16	124.73	4
Additive utility	2819.08	82.02	1	7255.28	128.9	2	7289.19	127.38	1
Proportional difference	3004.95	79.96	2	13401.67	126.94	11	9876.74	114.93	8
ITCH	3504.33	96.71	10	9336.58	165.12	9	14343.42	269.08	11
Tradeoff	8063.80	201.78	11	12068.47	295.06	10	14262.83	325.54	10

^6^

**Fig. 2:**
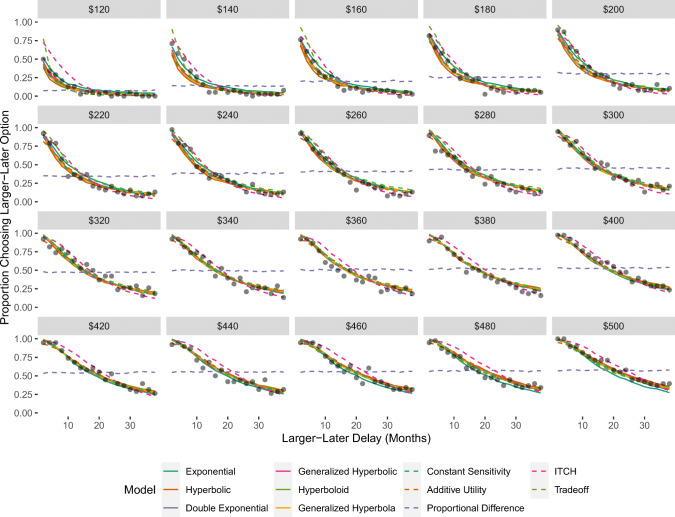
Proportion of choices in which the larger-later option was chosen as a function of delay and amount in the experiment used for Study 2. Observed proportions are represented by black dots. Predictions were generated based on the mean of the posterior predictive distribution

Figure 2 shows the fit of the models to the data that was used for Study 2. Participants in this experiment also became less likely to select the larger-later option as the delay (represented on the x-axis) increased, and became more likely to select the larger-later option as the magnitude (represented as the different panels) increased. As can be seen, 10 out of the 11 models provide very close fit to the data. The only model that cannot account for these results is the Proportional Difference model, which does not predict an effect of delay when one of the options offers an immediate reward. The models that performed best according to the WAIC were the Constant Sensitivity, Additive Utility, Generalized Hyperbolic, and Double Exponential models.

**Fig. 3:**
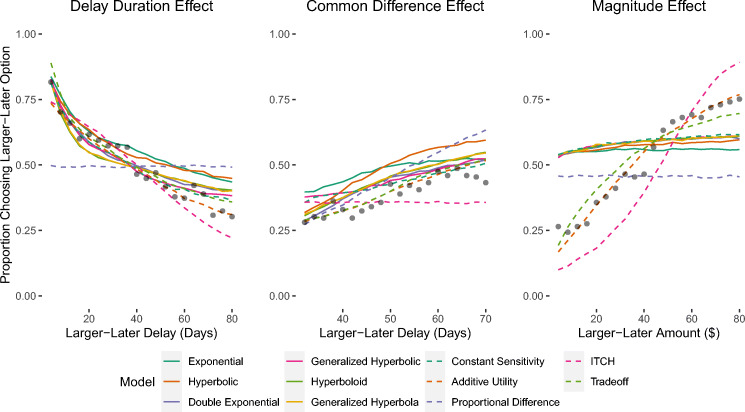
Proportion of choices in which the larger-later option was chosen in the experiment used for Study 2. Observed proportions are represented by black dots. Predictions were generated based on the mean of the posterior predictive distribution

Figure 3 shows the fit of the models to the data that was used for Study 3. The left panel shows the results for the choice set designed to elicit the delay duration effect. As can be seen, when the delay for the smaller-sooner option was always half the delay for the larger-later option, participants became less likely to select the larger-later option as the delays increased. Every model except the Proportional Difference model was able to capture this effect. The middle panel shows the results for the choice set designed to elicit the Common Difference effect. As can be seen, when the delay for the smaller-sooner option was always 30 days less than the delay for the larger-later option, participants became more likely to select the larger-later option as the delays increased. Every model except the ITCH model could reproduce this effect.

The right panel shows the results for the choice set designed to elicit the magnitude effect. As can be seen, when the amount associated with the smaller-sooner option was always half the amount associated with the larger-later option, participants become more likely to select the larger-later option as the amounts increased. Only the Additive Utility, Tradeoff, and (to a lesser extent) the ITCH models captured this effect. According to the WAIC results, the Additive Utility model performed best for Study 3.

These results combined suggest that, despite the close fit of most of the models in Studies 1 and 2, only a small number of models simultaneously captured the different behavioral regularities observed in Study 3. As expected, most of the discounted utility models failed to capture the magnitude effect. The Trade-off, ITCH, and Proportional Difference models also provided a le descss satisfactoryription of the data. At first glance, the poor performance of the attribute-wise models may appear inconsistent with prior research showing attribute-wise models to outperform discounted utility models (e.g., Amasino, Sullivan, Kranton, & Huettel, 2019; Dai & Busemeyer, 2014; Marzilli Ericson et al., 2015; Scholten et al., 2014). But the three attribute models demonstrated poor performance for very different reasons. The Trade-off model could account for the full pattern of results across the three studies, but the level of complexity the model employs to capture these results made it a less satisfactory explanation than the simpler models (at least as measured by the WAIC). It may be that the Tradeoff model only reliably outperforms simpler models when item sets are specifically designed to elicit more complex effects like gain-loss asymmetry, superadditivity, and subadditivity (e.g., Scholten et al.,, 2014).

The ITCH model was developed to capture the three effects observed in Study 3, and indeed may be able to reproduce these qualitative effects using a single set of parameter values (Marzilli Ericson et al., 2015). However, when we constrained parameters to have the same value across the subsets of items in Study 3, the ITCH model did not provide a good quantitative fit to all three effects simultaneously. This indicates that the ITCH may only be able to accurately capture these three behavioral regularities by assuming the parameter values vary across contexts. The Proportional Difference model performed poorly because it simply could not account for delay discounting when one option has an immediate reward (e.g., Study 2). When this is the case (i.e., when *t*_*SS*_ = 0), the second term in Eq. 10 reduces to $\frac {1}{1}$ regardless of the value of *t*_*LL*_, making the model unable to capture effects of delay. Nevertheless, the Additive Utility Model, which demonstrated the best descriptive adequacy, can be conceptualized as an attribute-wise model (see Footnote 3), specifically, a variant of the Weighted Additive Difference model from Dai & Busemeyer. This provides evidence, consistent with prior research, that certain attribute-wise models indeed outperform many discounted utility models.

Notably, the rank order of the models was fairly consistent across the three studies (particularly for Studies 2 and 3). The Additive Utility model in general demonstrated the best descriptive adequacy (it had the lowest WAIC value for Studies 1 and 3 and the second-lowest for Study 1) . This finding suggests that the unique additive relationship between the outcome value and the delay that is assumed by this model may be important for describing inter-temporal choices. By contrast, the ITCH and Tradeoff models were consistently among the poorest performing models.

## Parameter consistency

Prior to examining parameter recovery (see next section), we first consider the issue of parameter consistency. Parameter consistency refers to the extent to which a model’s parameter values are stable across contexts and is another important consideration when evaluating whether model parameters can be interpreted meaningfully. Parameters that change across contexts, experimental designs, or time are difficult to interpret in any absolute sense because their values depend on the exact settings in which the parameters were estimated.

For example, consider the case of discount rate, its relationship to the magnitude effect, and the Hyperbolic model. Technically, the concept of discount rate should be independent of variations along the dimension of money; according to rational models of inter-temporal choice, the same discount rate should apply to all decisions. However, in order to account for increased patience with rewards of higher magnitude, the Hyperbolic model can only assume different discount rates to explain the effect. This is, of course, not parsimonious (as there is an infinite number of magnitudes that might be considered) and indicates that discount rates in the Hyperbolic model are overly malleable and prone to changes in the experimental setting (e.g., those using different amounts of money). In other words, it may not provide stable measurements of an individual’s discounting behavior. Instead what is actually needed is a functional form that incorporates the effect of magnitude on discount rates for a suggested form, see Vincent (2016).

The Dai and Busemeyer (2014) dataset used in Study 3 provides a useful opportunity to examine parameter consistency. As described above, their dataset included three separate subsets of items each designed to elicit a different effect. As each participant completed all three subsets of items, we can estimate model parameters independently for each subset and compare them within participants. If model parameters are consistent within an individual, we would expect similar parameter estimates to be obtained when the same model is fit to each subset of items separately. If different parameter values are required to account for the different effects, this would suggest that the model’s parameters are more context dependent and therefore more difficult to interpret in a meaningful way.

We examine parameter consistency by analyzing the correlation between individual model parameters estimated based on the different subsets of items. Table 4 shows the 95% credible intervals on the correlations between estimates based on each pair of choice sets. As can be seen, the credible intervals for most correlations include zero, suggesting that it is plausible to assume there is no relationship between parameter estimates based on different subsets of items. Between the delay duration and common difference subsets of items (the DD / CD column), only five out of the 37 parameters examined had estimates that had credible correlations (shown in bold). Between the delay duration and magnitude effects, 9 out of 37 had credible correlations. Between the common difference and magnitude effects, 7 out of 37 had credible correlations. Across all 11 models, only two parameters had credible correlations for all three pairs of subsets. These were the *δ* parameter of the Proportional Difference model and the *β*_1_ parameter of the ITCH model (which both represent a bias towards sooner options). There was no model for which all parameters had credible correlations across all subsets of items. Overall, these results provide evidence against parameter consistency for most of the parameters considered.

**Table 4:** 95% credible intervals on the correlations between parameters estimated from three subsets of the Dai and Busemeyer (2014) data

Model	Parameter	DD / CD	DD / M	CD / M
Exponential	*k*	(− 0.21, 0.47)	**(0.02, 0.64**)	(− 0.24, 0.54)
	*σ*	(− 0.04, 0.56)	**(0.09, 0.56)**	(− 0.15, 0.42)
Hyperbolic	*k*	**(0.16, 0.78)**	(− 0.04, 0.45)	(− 0.07, 0.46)
	*σ*	**(0.14, 0.69)**	(− 0.01, 0.14)	(− 0.03, 0.21)
Double exponential	*β*	(− 0.27, 0.33)	(− 0.25, 0.35)	(− 0.25, 0.46)
	*δ*	(− 0.41, 0.63)	(− 0.16, 0.58)	(− 0.33, 0.35)
	*ω*	(− 0.12, 0.49)	(− 0.22, 0.38)	(− 0.27, 0.35)
	*σ*	(− 0.08, 0.49)	**(0.07, 0.53)**	(− 0.09, 0.55)
Generalized hyperbolic	*k*	(− 0.20, 0.37)	(− 0.20, 0.53)	(− 0.23, 0.39)
	*s*	**(0.16, 0.70)**	(− 0.24, 0.35)	(− 0.13, 0.41)
	*σ*	(− 0.04, 0.41)	**(0.06, 0.63)**	**(0.07, 0.63)**
Hyperboloid	*k*	(− 0.11, 0.53)	(− 0.17, 0.55)	(− 0.21, 0.47)
	*s*	(− 0.15, 0.49)	(− 0.26, 0.33)	(− 0.23, 0.40)
	*σ*	(− 0.06, 0.41)	**(0.07, 0.59)**	**(0.10, 0.66)**
Generalized hyperbola	*α*	(− 0.17, 0.53)	(− 0.18, 0.53)	(− 0.21, 0.46)
	*β*	(− 0.16, 0.59)	(− 0.01, 0.72)	(− 0.17, 0.52)
	*σ*	(− 0.07, 0.40)	**(0.07, 0.59)**	**(0.08, 0.65)**
Constant sensitivity	*α*	(− 0.03, 0.54)	(− 0.23, 0.41)	(− 0.27, 0.40)
	*β*	(− 0.23, 0.30)	(− 0.32, 0.27)	(− 0.19, 0.47)
	*σ*	(− 0.11, 0.49)	(− 0.05, 0.59)	**(0.05, 0.69)**
Additive utility	*α*	(− 0.11, 0.49)	**(0.18, 0.55)**	(− 0.26, 0.28)
	*β*	(− 0.07, 0.44)	(− 0.11, 0.40)	**(0.10, 0.61)**
	*λ*	(− 0.26, 0.40)	(− 0.24, 0.43)	(− 0.20, 0.46)
	*σ*	(− 0.06, 0.56)	(− 0.07, 0.54)	(− 0.11, 0.54)
Proportional difference	*δ*	**(0.43, 0.72)**	**(0.24, 0.60)**	**(0.19, 0.56)**
	*σ*	(− 0.20, 0.40)	(− 0.19, 0.44)	(− 0.15, 0.44)
ITCH	*β*_1_	**(0.00, 0.51)**	**(0.17, 0.55)**	**(0.02, 0.52)**
	*β*_*tA*_	(− 0.19, 0.54)	(− 0.29, 0.34)	(− 0.30, 0.37)
	*β*_*tR*_	(− 0.28, 0.39)	(− 0.31, 0.35)	(− 0.25, 0.42)
	*β*_*xA*_	(− 0.27, 0.38)	(− 0.15, 0.52)	(− 0.28, 0.36)
	*β*_*xR*_	(− 0.32, 0.34)	(− 0.30, 0.33)	(− 0.31, 0.34)
Tradeoff	*α*	(− 0.33, 0.33)	(− 0.33, 0.33)	(− 0.30, 0.35)
	*𝜖*	(− 0.45, 0.15)	(− 0.16, 0.40)	(− 0.30, 0.24)
	*γ*	(− 0.28, 0.38)	(− 0.29, 0.35)	(− 0.21, 0.37)
	*κ*	(− 0.28, 0.37)	(− 0.30, 0.34)	(− 0.05, 0.53)
	*τ*	(− 0.06, 0.50)	(− 0.21, 0.37)	(− 0.20, 0.43)
	*𝜃*	(− 0.37, 0.25)	(− 0.26, 0.38)	(− 0.33, 0.32)

Note: The DD / CD column contains the correlation between the parameters estimated from the delay duration and common difference subsets. The DD / M column contains the correlation between the estimates from the delay duration and magnitude subsets. The CD / M column contains the correlation between the estimates from the common difference and magnitude subsets. Credible intervals that do not include zero are shown in bold

## Parameter recovery

**Fig. 4:**
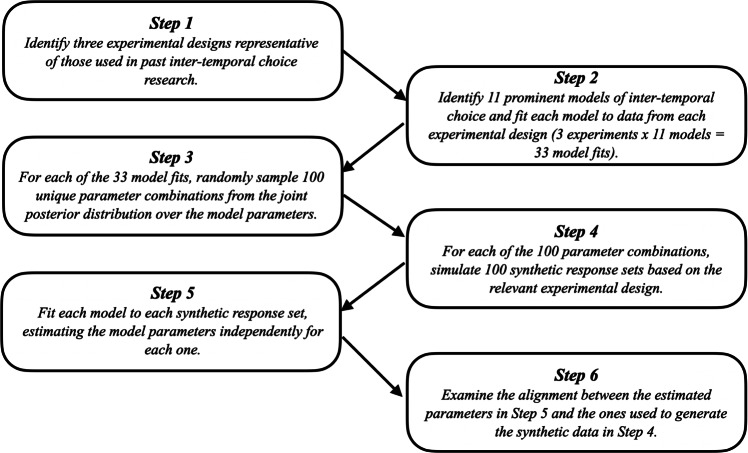
Summary of the approach used in the parameter recovery studies

A summary of the approach we use for the parameter recovery analysis is presented in Fig. 4. To evaluate the parameter recovery, we randomly sampled 100 parameter combinations per model from the posterior distributions obtained from fitting each model to each dataset. We did this by repeatedly sampling a participant from the dataset and then extracting that participant’s parameter values on a randomly sampled iteration. This method ensured that any dependencies between the parameters in each model would be preserved. For each parameter combination, we generated 100 sets of simulated responses using the same experimental design as the study to which the models were fit. We can think of this as a simulated participant making decisions according to a particular model under a particular parameter combination, completing the same experiment 100 times. For Study 3, where each participant responded to a different set of items, the simulated responses were generated using the choice set associated with the participant who provided the data-generating parameter values. For example, if the first combination of parameters was provided by participant *i*, participant *i*’s items would be used to generate the 100 sets of responses associated with that parameter combination. This method resulted in 10000 simulated response sets per model per experiment (100 for each parameter combination).

We then fit each model to the 10000 simulated response sets that it produced, estimating the model parameters independently for each set of responses (see Appendix B for model priors). This resulted in 3 experimental designs × 11 models × 100 data-generating parameter combinations × 100 response sets per parameter combination = 330000 response sets analyzed. For each parameter value examined, we interpreted the average 95% credible interval (CI) across the 100 simulated response sets as the measure of recovery. This measure allows us to examine the average precision with which any one participant’s parameters can be estimated.^7^

**Fig. 5:**
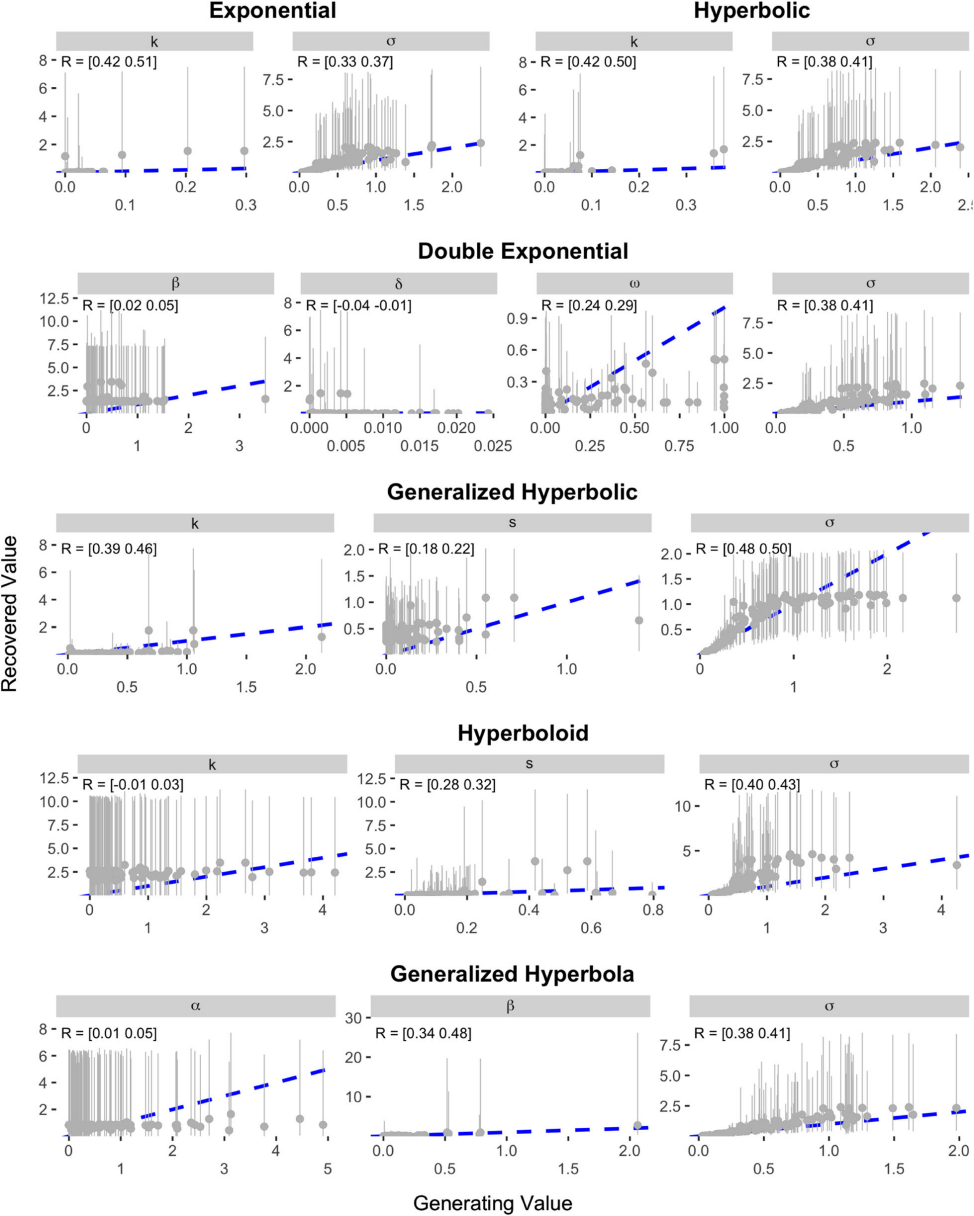
Results of parameter recovery analysis for the Exponential, Hyperbolic, Double Exponential, Generalized Hyperbolic, Hyperboloid, and Generalized Hyperbola models in Study 1. The x- and y-axes represent the generating and recovered parameter values respectively. Points represent the average posterior median value across simulated participants. Error bars represent the average 95% CI, which was calculated by taking the mean lower and upper bounds across simulated participants. The text in the top-left corner of each panel indicates the 95% CI on the correlation between the data-generating and recovered parameter values. The blue dotted line in each panel represents the identity line

**Fig. 6:**
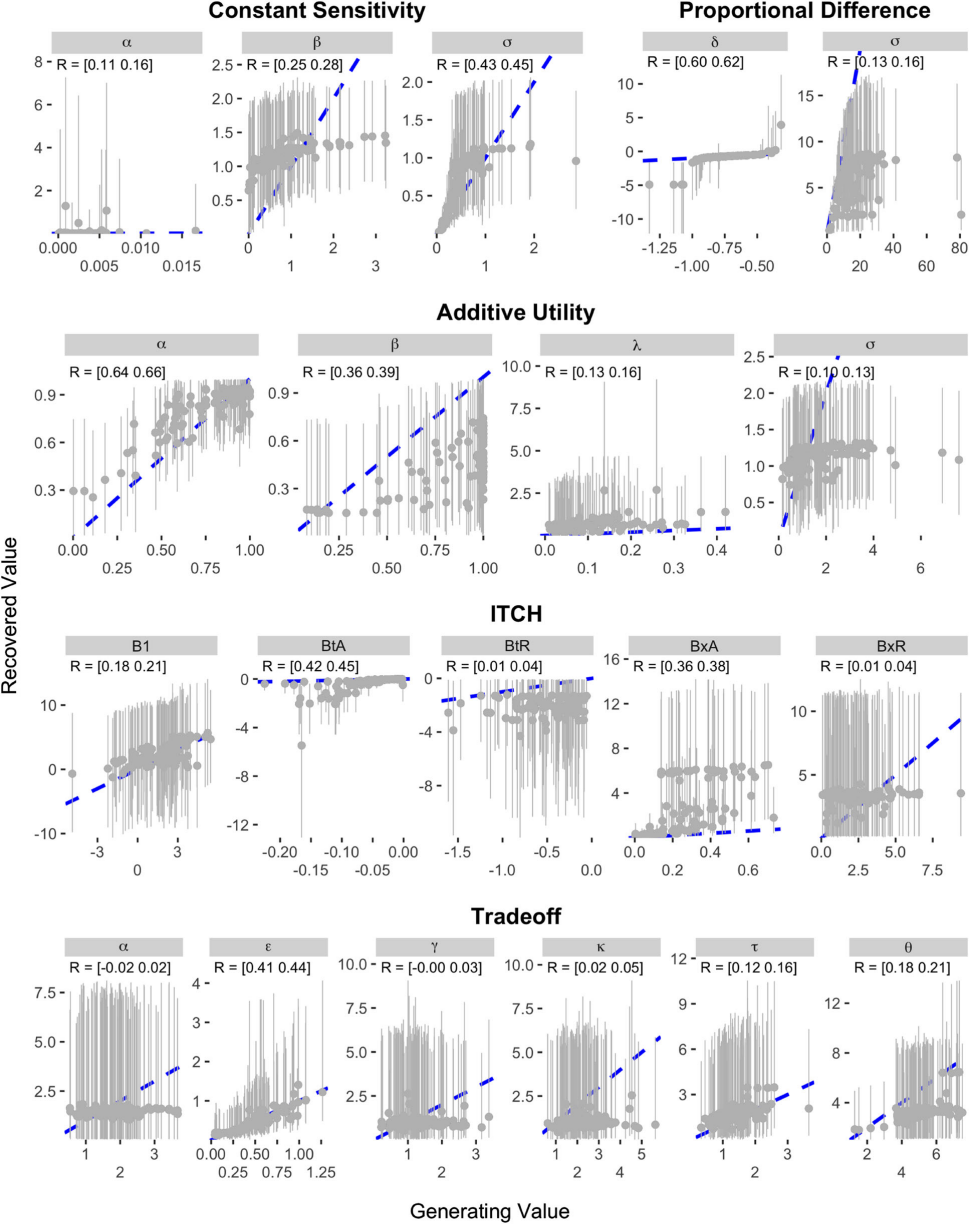
Results of parameter recovery analysis for the Constant Sensitivity, Proportional Difference, Additive Utility, ITCH, and Tradeoff models in Study 1. The x- and y-axes represent the generating and recovered parameter values respectively. Points represent the average posterior median value across simulated participants. Error bars represent the average 95% CI, which was calculated by taking the mean lower and upper bounds across simulated participants. The text in the top-left corner of each panel indicates the 95% CI on the correlation between the data-generating and recovered parameter values. The blue dotted line in each panel represents the identity line

The results of the recovery analysis for Study 1 are presented in Figs. 5 and 6. As can be seen, most of the models show rather poor recovery when parameters are estimated based on the Kirby et al. items. The Exponential and Hyperbolic models—the simplest models and therefore the ones that should be expected to demonstrate the best recovery—showed correlations between data-generating and recovered parameter values of between 0.33 and 0.51. These correlations show an association between the data-generating and recovered values, but are not high enough to inspire confidence that an estimated value accurately quantifies the true level of the underlying construct. The Generalized Hyperbolic, Constant Sensitivity, Proportional Difference, and Additive Utility models also showed correlations between data-generating and recovered parameter values that were all at least 0.1, but no correlation was higher than 0.66 (the *α* parameter in the Additive Utility model). The remaining five models all had at least one parameter with a generating-recovered value correlation as low as 0.02. Indeed, the Double Exponential, Hyperboloid, and Tradeoff models all contained at least one parameter in which the 95% credible interval on the correlation between data generating and recovered value included values that were negative.

**Fig. 7:**
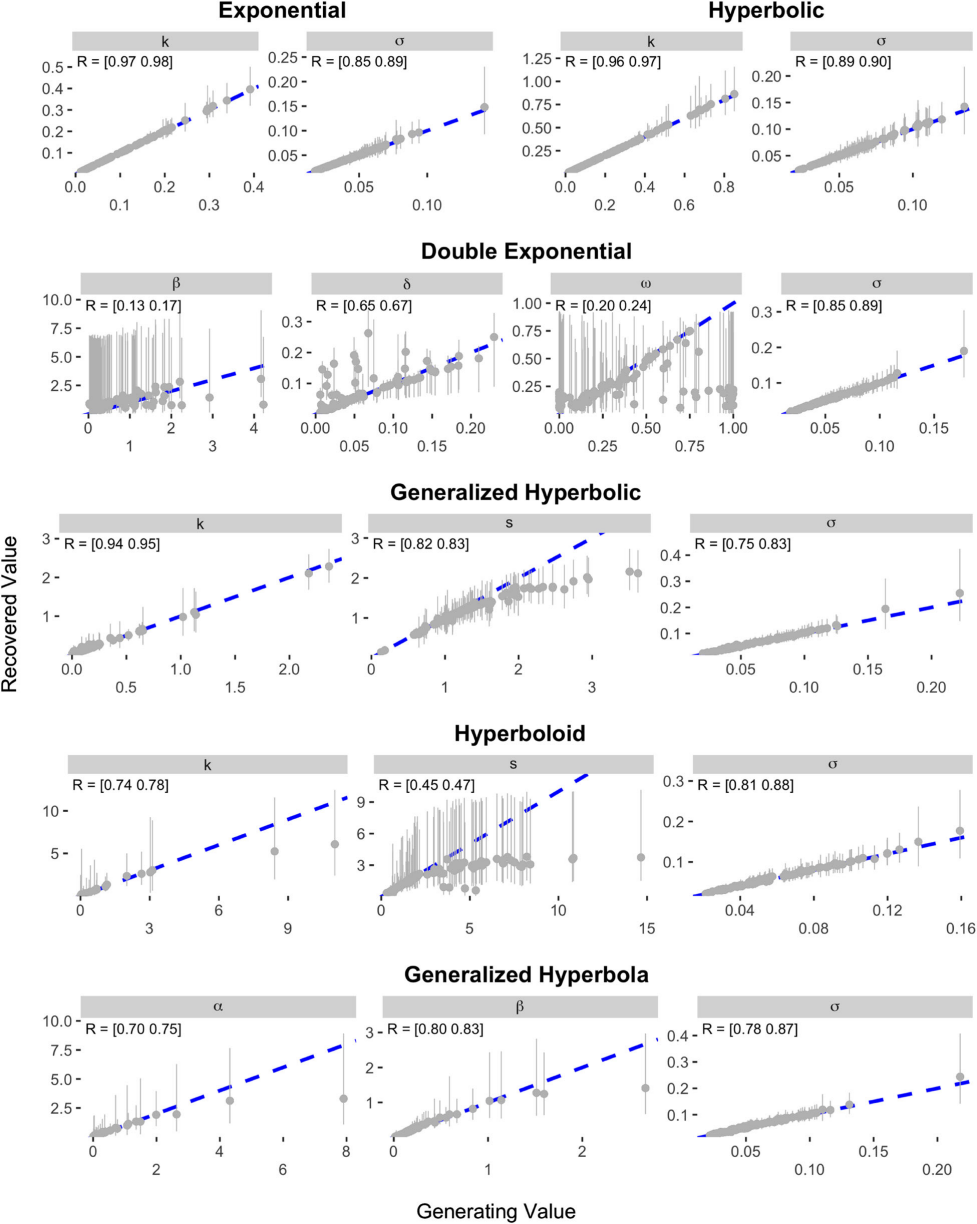
Results of parameter recovery analysis for the Exponential, Hyperbolic, Double Exponential, Generalized Hyperbolic, Hyperboloid, and Generalized Hyperbola models in Study 2. The x- and y-axes represent the generating and recovered parameter values respectively. Points represent the average posterior median value across simulated participants. Error bars represent the average 95% CI, which was calculated by taking the mean lower and upper bounds across simulated participants. The text in the top-left corner of each panel indicates the 95% CI on the correlation between the data-generating and recovered parameter values. The blue dotted line in each panel represents the identity line

**Fig. 8:**
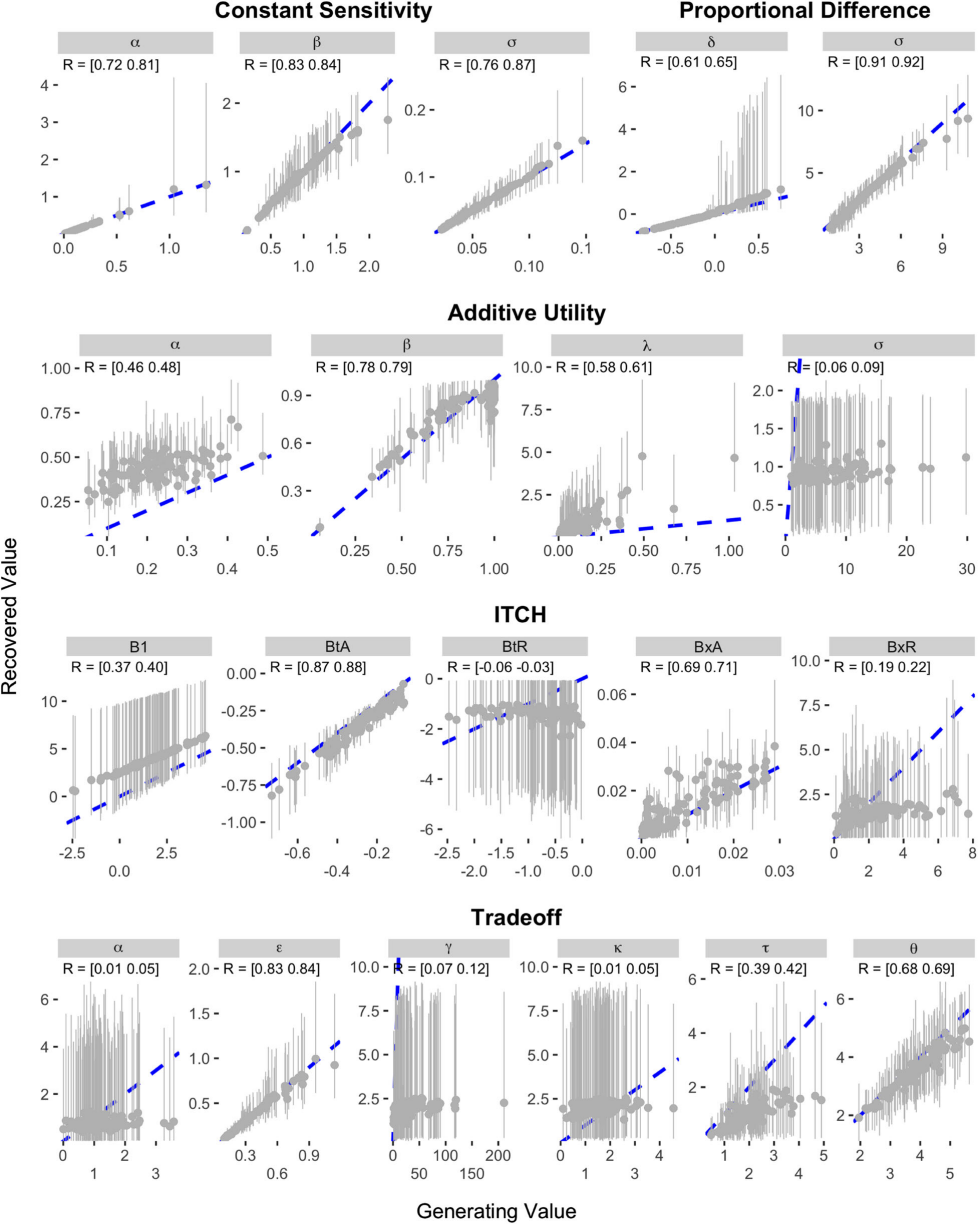
Results of parameter recovery analysis for the Constant Sensitivity, Proportional Difference, Additive Utility, ITCH, and Tradeoff models in Study 2. The x- and y-axes represent the generating and recovered parameter values respectively. Points represent the average posterior median value across simulated participants. Error bars represent the average 95% CI, which was calculated by taking the mean lower and upper bounds across simulated participants. The text in the top-left corner of each panel indicates the 95% CI on the correlation between the data-generating and recovered parameter values. The blue dotted line in each panel represents the identity line

The results of the recovery analysis for Study 2 are presented in Figs. 7 and 8. As can be seen, the parameter recovery was much stronger for the Konstantinidis et al. items than for the Kirby et al. items. In Study 2, the parameters of the Hyperbolic and Exponential models demonstrated the best recovery, with correlations between data-generating and recovered parameter values ranging between 0.85 and 0.98. The parameters of the Proportional Difference model were also well recovered, although estimates of the *δ* parameter were noisy when the data-generating value was positive. The next best models in terms of parameter recovery were the Generalized Hyperbolic, Generalized Hyperbola, and Constant Sensitivity models, which all had correlations between data-generating and recovered parameters that were above 0.7. Slightly worse was the Hyperboloid model, where the recovered values of the *s* parameter correlated only between 0.45 and 0.47 with the data-generating values. The remaining four models—the ITCH, Additive Utility, Double Exponential, and Tradeoff models—all demonstrated poorer recovery. All of these models except the Double Exponential had at least one parameter for which the correlation between data-generating and recovered values was less than 0.1.

**Fig. 9:**
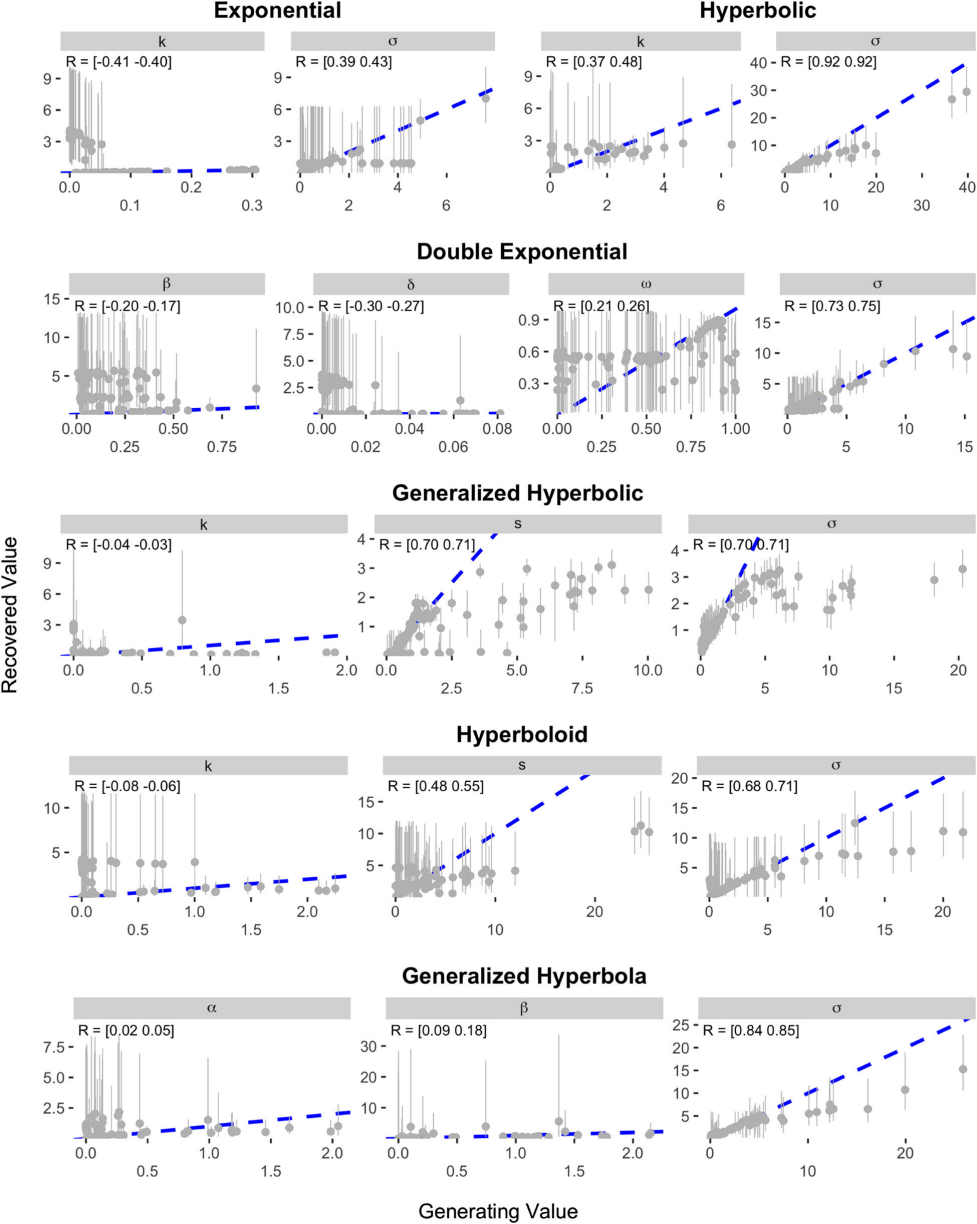
Results of parameter recovery analysis for the Exponential, Hyperbolic, Double Exponential, Generalized Hyperbolic, Hyperboloid, and Generalized Hyperbola models in Study 3. The x- and y-axes represent the generating and recovered parameter values respectively. Points represent the average posterior median value across simulated participants. Error bars represent the average 95% CI, which was calculated by taking the mean lower and upper bounds across simulated participants. The text in the top-left corner of each panel indicates the 95% CI on the correlation between the data-generating and recovered parameter values. The blue dotted line in each panel represents the identity line

**Fig. 10:**
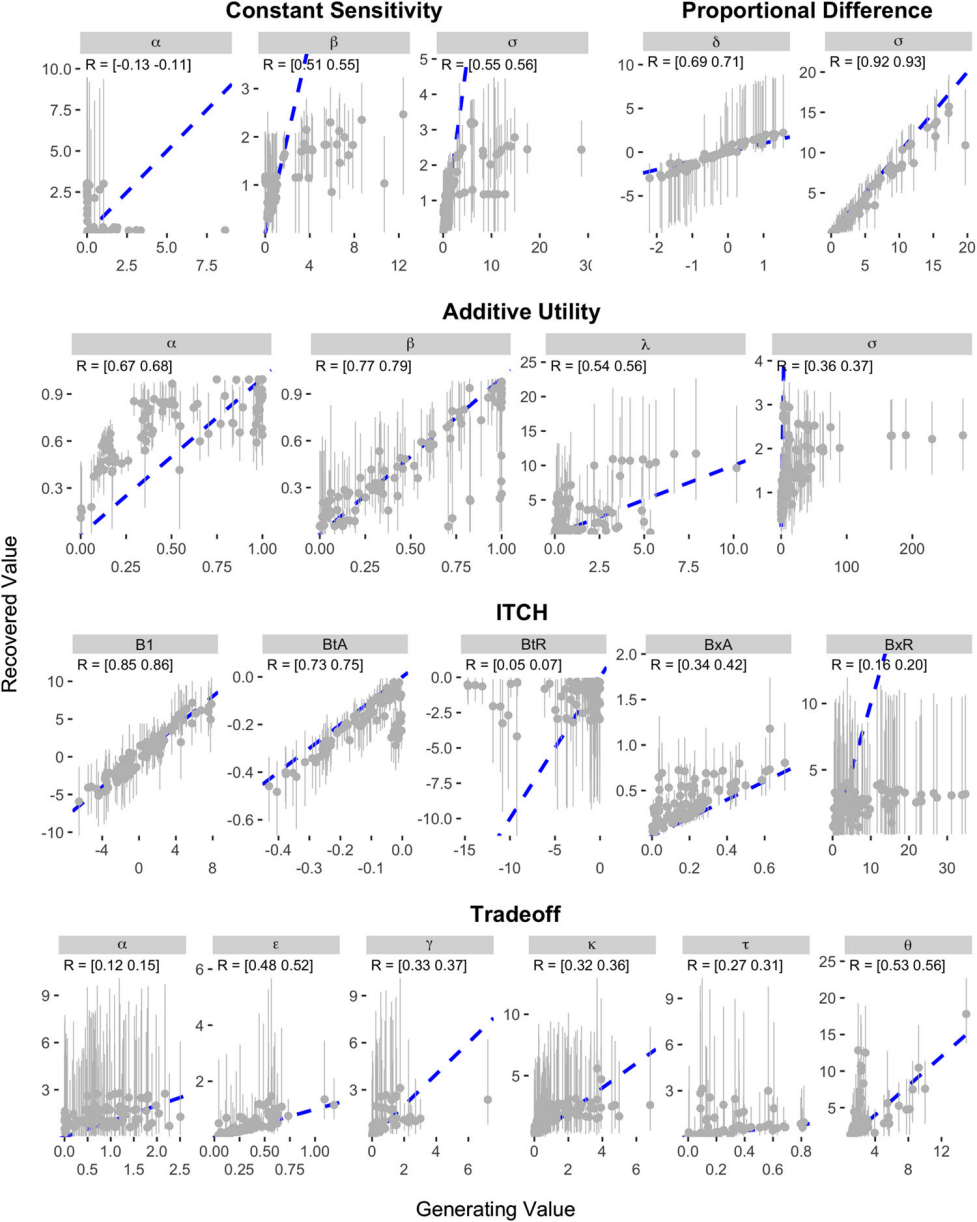
Results of parameter recovery analysis for the Constant Sensitivity, Proportional Difference, Additive Utility, ITCH, and Tradeoff models in Study 3. The x- and y-axes represent the generating and recovered parameter values respectively. Points represent the average posterior median value across simulated participants. Error bars represent the average 95% CI, which was calculated by taking the mean lower and upper bounds across simulated participants. The text in the top-left corner of each panel indicates the 95% CI on the correlation between the data-generating and recovered parameter values. The blue dotted line in each panel represents the identity line

The results of the recovery analysis for Study 3 are presented in Figs. 9 and 10. As can be seen, the parameter recovery was considerably worse for the design used in Study 3 compared to the one used in Study 2. The model that demonstrated the best parameter recovery in Study 3 was the Proportional Difference model, which was the only model to demonstrate better recovery in Study 3 than in Study 2. The other models that demonstrated strong recovery in Study 2—the Exponential, Hyperbolic, Hyperboloid, Generalized Hyperbolic, Generalized Hyperbola, and Constant Sensitivity models—all demonstrated surprisingly poor recovery in Study 3. These models, with the exception of the Hyperbolic model, all had at least one parameter for which the correlation between the data-generating and recovered parameter values was negative. In many cases, the parameter that was most poorly recovered was the most psychologically meaningful (e.g., *k* in the Exponential, Hyperbolic, Hyperboloid, and Generalized Hyperbolic; and *α* in the Generalized Hyperbola and Constant Sensitivity). The remaining four models also demonstrated poor recovery, with the Double Exponential being the poorest.

## Discussion

Models of inter-temporal choice have had widespread impact across many areas in management, organizational, behavioural and economic sciences as well as in neuroscience, clinical psychology and addiction studies. Though decades of work has been dedicated to developing models and evaluating their ability to account for established empirical effects, surprisingly little work has examined the extent to which model parameters can be reliably estimated and consequently used as meaningful descriptions of how people behave in the face of choices with delayed consequences. This is a problem, because estimation error can systematically bias the conclusions that are drawn from parameter estimates. Given that inter-temporal choice models are often used for measurement purposes, it is important to determine the reliability with which each model’s parameters can be estimated.

To address this problem, we examined the reliability of the parameters estimated from 11 prominent models of inter-temporal choice. To do this, we first fit each model to data from three inter-temporal choice experiments to identify plausible combinations of parameters in each design. We then conducted a parameter recovery analysis, in which we generated data from each model using randomly sampled parameter combinations and examined the correspondence between the estimated and data-generating parameter values when each model was fit to the simulated/synthetic data. We then examined the stability in each models’ parameter estimates by assessing the consistency in estimated values for the same individual across three different sets of items.

We found that the ability to recover model parameters varied widely among the 11 models and depended on the experimental design. In Study 2, where all participants received the same 380 items, recoverability declined with the number of model parameters. The three 2-parameter models—the Exponential, Hyperbolic, and Proportional Difference—all have parameters that were well recovered (except for the *δ* parameter in the proportional difference model when its true value is positive). The four 3-parameter models—the Hyperboloid, Generalized Hyperbolic, Generalized Hyperbola, and Constant Sensitivity models—had somewhat poorer recoverability. The parameter estimates of the remaining models were much more difficult to recover.

We also found that the items on which parameter estimates are based have a strong impact on parameter recovery. In Study 1, in which participants responded to the 27 items introduced by Kirby, Petry, and Bickel (1999), recovery was generally poorer than in Study 2. The likely explanation for this is that this choice set contained too few items and therefore provided insufficient information to identify the models’ parameters. In Study 3, where each participant had a unique set of items that was specifically tailored to generate specific empirical effects, recovery was also generally poorer than in Study 2. This was the case even though the set of items used in Study 3 was similar in size to the set used in Study 2 (380 in Study 2 versus 300 in Study 3). One reason for the poor parameter recovery in Study 3 may have been that the item generation procedure used in this design resulted in rather polarized decision-making patterns. For the subset of items used for the delay duration and common difference effects, the majority of participants chose the same option at least 85% of the time. This lack of variance in choice selection flattens the likelihood surface for many models, making it harder to differentiate between the plausibility of different combinations of parameter values. The choice set used in Study 2, by contrast, produced much more varied responding, with most participants regularly utilizing both the larger-later and smaller-sooner options.

We also found a lack of consistency in the parameter estimates between the three subsets of items used in Study 3. There was no model for which participants’ parameter estimates had credible correlations across all subsets of items. On the surface, these results suggest that all 11 models have parameters whose value changes depending on the context, and therefore cannot be interpreted in an absolute sense. On one hand, this finding suggests that certain model parameters may have issues that extend beyond poor recovery. The lack of association among participant’s parameter values across contexts raises questions about the validity of these estimates. On the other hand, given the recovery of the parameter estimates that were based on the items used in Study 3, this result should be interpreted with caution. It is possible that the apparent lack of consistency across choice sets may simply be the result of the unreliability of those estimates or correlation between them (Vincent & Stewart, 2020) as opposed to a genuine change in the constructs they represent. To make stronger conclusions about parameter consistency, we need to examine it in a context in which the parameters can be reliably estimated.

### Recommendations

Based on our results, the approach that we believe is best suited for measurement is to estimate the parameters of the Exponential and Hyperbolic models based on data generated using items similar to those used in Study 2. The parameters of these models were among the most reliably estimated in Study 2. However, as these models only capture the delay duration and common difference effects (in the latter case), this should only be done when other effects are neither relevant to the behaviour being measured, nor present in the item-set. For example, if, as was the case in Study 3, large magnitude effects are present, the discount rate (i.e., *k*) parameters of the Exponential and Hyperbolic models will be less meaningful. The parameters of the Proportional Difference model were also highly well-recovered. However, we cannot recommend the use of this model for measurement due to concerns about the validity of the model as a plausible explanation of inter-temporal choice (in addition to failing to capture basic empirical trends, such as the delay duration effect, this model is also incapable of accounting for behaviour when one of the items does not have a delay).

We urge extreme caution when considering parameter estimates that are based on the Kirby et al. items. As shown in Study 1, parameter estimates based on these items were noisy in all of the 11 models we examined, suggesting that they may not provide a reliable index of the underlying construct they are meant to represent when estimated in this way. We also urge caution when considering parameter estimates derived from designs such as the one used in Dai and Busemeyer (2014) in which items are tailored to participants in order to elicit specific effects. Study 3 showed that this design also resulted in unreliable parameter estimates. Until we know more about how such item-tailoring procedures affect the parameters estimated from models fit to the resulting data, we believe parameters estimated from such choice sets should not be used for measurement purposes.

The most important recommendation we can make based on our findings, therefore, is that parameter recovery needs to be assessed for the specific design that will be used for parameter estimation. The reliability of a model’s parameter estimates depends to a large extent on the information upon which parameter estimates are based. This can be seen most clearly by the fact that the ability to recover the *k* parameter from the Exponential and Hyperbolic models was high in Study 2, lower in Study 1, and disappeared in Study 3. It is dangerous to assume that because a model’s parameters were reliably recovered using a particular design, those parameter estimates can always be trusted. If one wishes to interpret parameters based on a different set of items to those considered here, the onus is on the researcher to first establish the reliability of those parameter estimates.

### Additional considerations and future work

It is important to note that the analysis conducted here does not constitute an exhaustive assessment of all possible models of inter-temporal choice or methods for estimating their parameters. While we believe the three choice sets we considered here are representative of the types of choice sets that are typically used to study inter-temporal choice, there may be other sets of items for which parameter recovery is stronger. One useful next step for those wishing to use the models of inter-temporal choice that demonstrated poorer recovery in our analysis for measurement purposes would be to work towards identifying or developing choice sets that produce more reliable parameter estimates for these models. One way to do this would be to simulate the model under a variety of parameter settings and identify choices for which changing the parameter value(s) changes the decision predicted by the model. This allows the researcher to pinpoint the items that are most diagnostic for the parameters being estimated.

Hierarchical modelling also offers a promising tool that may further facilitate reliable parameter estimation. Molloy et al., (2020) demonstrated that a hierarchical Bayesian framework can increase the reliability of estimates of the parameters of the hyperbolic model, because it allows information about the parameters o be pooled across participants. Such a framework is likely to facilitate parameter estimation for other models as well. Re-parameterizing models may also help overcome estimation challenges. Krefeld-Schwalb, Pachur, and Scheibehenne (2022) showed that treating model parameters themselves as stochastic, as opposed to assuming noise originates in the choice process, can decrease intercorrelations between parameters. Specifying inter-temporal choice models in this way may therefore improve the reliability of parameter estimates. That said, more complex models such as the Trade-off model may require additional steps to be taken before its parameters can be reliably estimated. For these models, it may be necessary to fix certain parameters to plausible values in advance. For example, if the researcher is not particularly interested in quantifying the level of superadditivity in the choice process, they may consider fixing the *𝜃* parameter to one (i.e., no superadditivity) or some other plausible value (if appropriate). This would provide further constraint to the model that would facilitate the estimation of the other parameters.

Although we believe the data-generation procedures examined here are representative of the methods that are commonly used to estimate inter-temporal choice model parameters, there are other approaches that warrant consideration in future work. One such method is the adaptive design optimization approach (Cavagnaro, Gonzalez, Myung, & Pitt, 2013; Toubia, Johnson, Evgeniou, & Delquie, 2013; Vincent & Rainforth, 2017; Yang, Pitt, Ahn, & Myung, 2021). Adaptive design optimization involves updating parameter estimates in real time and generating items on the fly that maximize information gain. This method has the potential to deliver more precise information about model parameters, because it tailors the items to make them maximally diagnostic of model parameters. Adaptive design optimization may therefore yield more reliable parameter estimates, making it possible to use a wider range of models for measurement purposes.

One class of models that we did not examine is dynamic accounts of inter-temporal choice, which are based on evidence accumulation models (see Dai & Busemeyer, 2014; Dai et al., 2018; Konstantinidis et al., 2020; Rodriguez et al., 2014). These models describe the dynamic process by which preferences for the possible options evolve over time until preference for one option becomes strong enough that it is selected. They predict not only the choices that people make but also the time it takes to make those choices. These dynamic models therefore have the potential to utilize more information for parameter estimation, which may enable parameters to be more reliably estimated when models are implemented within this framework. Previous research that has examined these models has typically focused more on model comparison than parameter estimation (e.g., (Dai & Busemeyer, 2014); (Dai, Pleskac, & Pachur, 2018)). However, these models may provide a useful next step for those wishing to develop better calibrated tools for measuring components of the inter-temporal choice process.

One source of (un)reliability in model parameters that was not examined here was the parameters’ (in)stability over time. Although Study 3 showed variability within participants in parameters estimated from different sets of items, we attributed these differences to the choice context rather than to endogenous variability in process components. But how similar are a participant’s parameter estimates when the same individual completes the same choice set at different times? Studies examining the test-retest reliability of measures of delay discounting have shown evidence of stability over time when participants complete the same choice set (e.g., Anokhin, Golosheykin, & Mulligan, 2015; Kirby, 2009; Simpson & Vuchinich, 2000). Yet we also know that preference can (but do not always) change over time as outcomes draw nearer (Read, Frederick, & Airoldi, 2012). Moreover, the question remains as to the stability of other process components quantified by models of inter-temporal choice beyond delay discounting. Once we develop models and paradigms that produce well-recovered parameters, we can work toward answering such questions about temporal stability.

As a final point, we believe it is important to clarify that the failure to recover a model’s parameters does not mean that the model in question is not a plausible description of inter-temporal choice. In other words, parameter recovery is not a necessary condition for descriptive adequacy. Indeed, attribute-wise accounts such as the Tradeoff model may provide a more complete description of the behavioral regularities observed in inter-temporal choice than many of their alternative-wise predecessors (Scholten et al., 2014). However, the poor recovery observed for the Trade-off and other models does mean that their parameter estimates cannot be mapped meaningfully to underlying processes, which we believe is an important factor that contributes to a model’s usefulness.

### Conclusion

Decades of research has been dedicated to the development and testing of inter-temporal choice models. However, the lack of work examining the reliability of these models’ parameter estimates calls into question conclusions that have been made based on the assumption that parameter estimates map meaningfully onto latent components of the inter-temporal choice process. Our findings suggest that many of the parameter estimates reported in previous research are likely unreliable, and that caution should therefore be used in making inferences based on their values. Researchers wishing to do so must first establish that the model’s parameters can be reliably estimated given the experimental protocol that will be used to generate the data and the particular parameterization of the model being used. Models of inter-temporal choice have the potential to yield important insights into the process by which humans evaluate outcomes that are temporally distant, but care must be taken to ensure such insights are robust.

## Appendix A: Priors for hierarchical models used to assess descriptive adequacy

**Table:**

Model	Parameter	Population Parameters	
Exponential	$k \sim \ Gamma(A,B)$	$A \sim \ Normal_{+}(0,1) $	$B \sim \ Normal_{+}(0,1) $
	$\sigma \sim \ Gamma(A,B)$	$A \sim \ Normal_{+}(0,1) $	$B \sim \ Normal_{+}(0,1) $
Hyperbolic	$k \sim \ Gamma(A,B)$	$A \sim \ Normal_{+}(0,1) $	$B \sim \ Normal_{+}(0,1) $
	$\sigma \sim \ Gamma(A,B)$	$A \sim \ Normal_{+}(0,1) $	$B \sim \ Normal_{+}(0,1) $
Double	$\omega \sim \ Beta(A,B)$	$L \sim \ Uniform(0,1) $	$S \sim \ Gamma(1,20) $
Exponential	${\Theta } \sim \ Gamma(A,B)$	$A \sim \ Normal_{+}(0,1) $	$B \sim \ Normal_{+}(0,1) $
	$\delta \sim \ Gamma(A,B)$	$A \sim \ Normal_{+}(0,1) $	$B \sim \ Normal_{+}(0,1) $
	$\sigma \sim \ Gamma(A,B)$	$A \sim \ Normal_{+}(0,1) $	$B \sim \ Normal_{+}(0,1) $
Generalized	$k \sim \ Gamma(A,B)$	$A \sim \ Normal_{+}(0,1) $	$B \sim \ Normal_{+}(0,1) $
Hyperbolic	$s \sim \ Gamma(A,B)$	$A \sim \ Normal_{+}(0,1) $	$B \sim \ Normal_{+}(0,1) $
	$\sigma \sim \ Gamma(A,B)$	$A \sim \ Normal_{+}(0,1) $	$B \sim \ Normal_{+}(0,1) $
Hyperboloid	$k \sim \ Gamma(A,B)$	$A \sim \ Normal_{+}(0,1) $	$B \sim \ Normal_{+}(0,1) $
	$s \sim \ Gamma(A,B)$	$A \sim \ Normal_{+}(0,1) $	$B \sim \ Normal_{+}(0,1) $
	$\sigma \sim \ Gamma(A,B)$	$A \sim \ Normal_{+}(0,1) $	$B \sim \ Normal_{+}(0,1) $
Generalized	$\alpha \sim \ Gamma(A,B)$	$A \sim \ Normal_{+}(0,1) $	$B \sim \ Normal_{+}(0,1) $
Hyperbola	${\Theta } \sim \ Gamma(A,B)$	$A \sim \ Normal_{+}(0,1) $	$B \sim \ Normal_{+}(0,1) $
	$\sigma \sim \ Gamma(A,B)$	$A \sim \ Normal_{+}(0,1) $	$B \sim \ Normal_{+}(0,1) $
Constant	$\alpha \sim \ Gamma(A,B)$	$A \sim \ Normal_{+}(0,1) $	$B \sim \ Normal_{+}(0,1) $
Sensitivity	$\beta \sim \ Gamma(A,B)$	$A \sim \ Normal_{+}(0,1) $	$B \sim \ Normal_{+}(0,1) $
	$\sigma \sim \ Gamma(A,B)$	$A \sim \ Normal_{+}(0,1) $	$B \sim \ Normal_{+}(0,1) $
Additive	$\alpha \sim \ Beta(A,B)$	$L \sim \ Uniform(0,1) $	$S \sim \ Gamma(1,20) $
Utility	$\beta \sim \ Beta(A,B)$	$L \sim \ Uniform(0,1) $	$S \sim \ Gamma(1,20) $
	$\lambda \sim \ Gamma(A,B)$	$A \sim \ Normal_{+}(0,1) $	$B \sim \ Normal_{+}(0,1) $
	$\sigma \sim \ Gamma(A,B)$	$A \sim \ Normal_{+}(0,1) $	$B \sim \ Normal_{+}(0,1) $
Proportional	$\delta \sim \ Normal(L,S)$	$L \sim \ Normal(0,1) $	$S \sim \ Normal_{+}(0,1) $
Difference	$\sigma \sim \ Gamma(A,B)$	$A \sim \ Normal_{+}(0,1) $	$B \sim \ Normal_{+}(0,1) $
ITCH	$\beta _{1} \sim \ Normal(L,S)$	$L \sim \ Normal(0,1) $	$S \sim \ Normal_{+}(0,1) $
	$\beta _{xA} \sim \ Normal_{+}(L,S)$	$L \sim \ Normal_{+}(0,1) $	$S \sim \ Normal_{+}(0,1) $
	$\beta _{xR} \sim \ Normal_{+}(L,S)$	$L \sim \ Normal_{+}(0,1) $	$S \sim \ Normal_{+}(0,1) $
	$\beta _{tA} \sim \ Normal_{-}(L,S)$	$L \sim \ Normal_{-}(0,1) $	$S \sim \ Normal_{+}(0,1) $
	$\beta _{tR} \sim \ Normal_{-}(L,S)$	$L \sim \ Normal_{-}(0,1) $	$S \sim \ Normal_{+}(0,1) $
Tradeoff	$\gamma \sim \ Gamma(A,B)$	$A \sim \ Normal_{+}(0,1) $	$B \sim \ Normal_{+}(0,1) $
	$\tau \sim \ Gamma(A,B)$	$A \sim \ Normal_{+}(0,1) $	$B \sim \ Normal_{+}(0,1) $
	${\Theta } \sim \ Gamma(A,B)$	$A \sim \ Normal_{+}(0,1) $	$B \sim \ Normal_{+}(0,1) $
	$\kappa \sim \ Gamma(A,B)$	$A \sim \ Normal_{+}(0,1) $	$B \sim \ Normal_{+}(0,1) $
	$\alpha \sim \ Gamma(A,B)$	$A \sim \ Normal_{+}(0,1) $	$B \sim \ Normal_{+}(0,1) $
	$\epsilon \sim \ Gamma(A,B)$	$A \sim \ Normal_{+}(0,1) $	$B \sim \ Normal_{+}(0,1) $

Note: *N**o**r**m**a**l*_+_(*L*,*S*) represents a truncated normal distribution with mean *L*, standard deviation *S*, a lower bound of 0, and no upper bound. *N**o**r**m**a**l*_−_ represents a truncated normal distribution with an upper bound of 0 and no lower bound. *G**a**m**m**a*(*A*,*B*) represents a gamma distribution with shape *A* and scale *B*. Certain models were reparameterized for efficiency reasons and/or in order to enforce parameter constraints. In the double exponential model, *β* = Θ + *δ*. In the generalized hyperbola model, the parameter *β* = Θ × *α*. For the additive utility and double exponential models, *A* = *L* × *S* and *B* = (1 − *L*) × *S*. For the tradeoff model, *𝜃* = Θ + 1

## Appendix B: Priors for models used to assess parameter recovery

**Table:**

Model	Parameter
Exponential	$k \sim \ Gamma(1,0.5)$
	$\sigma \sim \ Gamma(1,0.5)$
Hyperbolic	$k \sim \ Gamma(1,0.5)$
	$\sigma \sim \ Gamma(1,0.5)$
Double	$\omega \sim \ Uniform(0,1) $
Exponential	${\Theta } \sim \ Gamma(1,0.5)$
	$\delta \sim \ Gamma(1,0.5)$
	$\sigma \sim \ Gamma(1,0.5)$
Generalized	$k \sim \ Gamma(1,0.5)$
Hyperbolic	$s \sim \ Gamma(1,0.5)$
	$\sigma \sim \ Gamma(1,0.5)$
Hyperboloid	$k \sim \ Normal_{+}(0,5)$
	$s \sim \ Normal_{+}(0,5)$
	$\sigma \sim \ Normal_{+}(0,5)$
Generalized	$\alpha \sim \ Gamma(1,0.5)$
Hyperbola	${\Theta } \sim \ Gamma(1,0.5)$
	$\sigma \sim \ Gamma(1,0.5)$
Constant	$\alpha \sim \ Gamma(1,0.5)$
Sensitivity	$\beta \sim \ Gamma(1,0.5)$
	$\sigma \sim \ Gamma(1,0.5)$
Additive	$\alpha \sim \ Uniform(0,1)$
Utility	$\beta \sim \ Uniform(0,1)$
	$\lambda \sim \ Gamma(1,0.5)$
	$\sigma \sim \ Gamma(1,0.5)$
Proportional	$\delta \sim \ Normal(0,5)$
Difference	$\sigma \sim \ Gamma(1,0.5)$
ITCH	$\beta _{1} \sim \ Normal(0,5)$
	$\beta _{xA} \sim \ Normal_{+}(0,5)$
	$\beta _{xR} \sim \ Normal_{+}(0,5)$
	$\beta _{tA} \sim \ Normal_{-}(0,5)$
	$\beta _{tR} \sim \ Normal_{-}(0,5)$
Tradeoff	$\gamma \sim \ Gamma(1,0.5)$
	$\tau \sim \ Gamma(1,0.5)$
	${\Theta } \sim \ Gamma(1,0.5)$
	$\kappa \sim \ Gamma(1,0.5)$
	$\alpha \sim \ Gamma(1,0.5)$
	$\epsilon \sim \ Gamma(1,0.5)$

Note: *N**o**r**m**a**l*_+_(*L*,*S*) represents a truncated normal distribution with mean *L*, standard deviation *S*, a lower bound of 0, and no upper bound. *G**a**m**m**a*(*A*,*B*) represents a gamma distribution with shape *A* and scale *B*. Certain models were reparameterized for efficiency reasons and/or in order to enforce parameter constraints. In the double exponential model, *β* = Θ + *δ*. In the generalized hyperbola model, the parameter *β* = Θ × *α*. For the additive utility and double exponential models, *A* = *L* × *S* and *B* = (1 − *L*) × *S*. For the tradeoff model, *𝜃* = Θ + 1

## Footnotes

1 The discount rate has a different meaning than the discount factor. The discount rate refers to the degree to which future rewards or outcomes are discounted, whereas the discount factor refers to the discounted value of the reward or outcome (see Laibson, 2003).

2 While the exponential model does not account for the common difference or magnitude effects, combining it with the logistic function does lead to preference strength changing as a function of outcome magnitude and common delay. Multiplying both outcomes by a common factor, similarly multiplies their absolute difference in discounted utilities by the same factor, without changing the sign of this difference. This means that the magnitude effect leads to stronger preferences, i.e. those who prefer the SS (LL) option for small amounts prefer the SS (LL) option more strongly for large amounts. Conversely adding a common delay weakens preferences, without reversing them, as discounting both options over an extra time period results in both having their discounted utility, and therefore the difference, decreased by the same additional proportion. Similar changes in preference strength occur for the other discounted utility models.

3 There are also non-linear utility functions that can allow the discounting models to account for the magnitude effect, in particular those with increasing elasticity, whereby multiplying both outcomes by a constant leads to a larger proportional change in the value of the larger outcome (Loewenstein & Prelec, 1992; Scholten & Read, 2010). However, in addition to these transformations rarely being employed in the literature, it is also an open question as to whether the magnitude effect is best attributed to properties of the value function, or to features of the discount rate, such as proposed by (Vincent, 2016).

4 In our framework, because we assume choice is based on the difference in discounted utilities, the Additive Utility model can also be conceptualised as an attribute-wise model. The utility difference produced by the model can be re-arranged into a difference between attribute differences, i.e., $d = (x_{LL}^{\alpha } - x_{SS}^{\alpha }) - \lambda (t_{LL}^{\beta }-t_{SS}^{\beta })$. As this is passed to a logistic function to calculate the choice probability, the additive utility model is equivalent to a version of the *Weighted Additive Difference model with direct differences* (Dai & Busemeyer, 2014).

5 We thank Daniel Read and Junyi Dai for making their data available.

6 We also compared the models using the leave-one-out cross-validation information criterion (Vehtari, Gelman, & Gabry, 2017). The model rank order was identical to the results according to the WAIC, so we only report the WAIC results here.

7 An alternative approach would be to assess parameter recovery using a hierarchical model in which the simulated response sets are assumed to be drawn from a common population and interpret the population mean to assess recovery. The problem with this approach, however, is that the precision with which the population mean is estimated depends on the number of simulated response sets. Additionally, the ability to recover the population mean does not necessarily indicate that the parameters of any individual participant will themselves be recovered.
